# Selective Paste Intrusion—Shadowing Effects from Rebar Protrusion in the Particle Bed and Their Impact on Bond Strength

**DOI:** 10.3390/ma19183954

**Published:** 2026-09-17

**Authors:** Alexander Straßer, Thomas Kränkel, Christoph Gehlen

**Affiliations:** Chair of Materials Science and Testing, Centre for Building Materials (cbm), School of Engineering and Design, Technical University of Munich, 85748 Garching, Germany; thomas.kraenkel@tum.de (T.K.);

**Keywords:** Selective Paste Intrusion, SPI, Wire Arc Additive Manufacturing, WAAM, additive manufacturing, bond behaviour, reinforcement integration, shadowing, particle bed, push-through test

## Abstract

Integrating Wire Arc Additive Manufacturing (WAAM) into the Selective Paste Intrusion (SPI) process enables the fully additive fabrication of reinforced concrete structures with complex geometries. Previous investigations have demonstrated that the thermal impact of the WAAM process can adversely affect the SPI process. Thus, dedicated cooling strategies are required. One proposed approach increases the vertical distance between the welding point and the particle bed by introducing a defined vertical protrusion of the reinforcement bar. This configuration may give rise to shadowing effects, here understood as a process-induced disturbance of material deposition in the vicinity of the protruding bar. Two distinct manifestations are considered in parallel. The first is a geometrically projected shadowed region within the particle bed, depending on bar diameter and inclination. The second is a layer-wise modification of the contact zone along the lower half of the bar surface within the bond length, largely independent of inclination. To isolate the geometric component from thermal effects, the present study focuses on controlled reinforcement configurations with constant vertical protrusion. Two hypotheses are tested: bond decreases with increasing bar diameter (H1), and, at constant diameter, with decreasing inclination angle (H2), the latter being the signature of the projected shadow. To assess these effects, reinforcement bars with a constant vertical protrusion of 40 mm and varying inclination angles were embedded into the particle bed, and concrete specimens were produced above them using the SPI process. Bar diameters of 8 mm, 16 mm, and 25 mm and inclination angles from 0° to 90° in 15° increments were investigated systematically. Bond strength was determined using push-through tests derived from RILEM RC6, and the bond response was evaluated against both a quantitative measure of the projected shadowed area and a process-based indicator of the affected contact zone. The bar diameter dominates the bond response, most pronounced at the developed-interlock and capacity levels. The inclination angle produces no monotonic trend from 0° to 90°, and individual angle contrasts remain largely within the experimental scatter. The projected shadowed area cannot consistently explain the observed behaviour and is at most a secondary factor, whereas the layer-wise contact-zone disturbance along the lower bar surface is the most probable interpretation of the data. The findings identify shadowing as a boundary condition for reinforcement integration in SPI: the observed bond reduction at the developed-interlock and capacity levels is attributed to the layer-wise contact-zone disturbance rather than to the projected shadowed area, an attribution that remains a hypothesis until the contact zone has been verified directly.

## 1. Introduction

**Figure 1:**
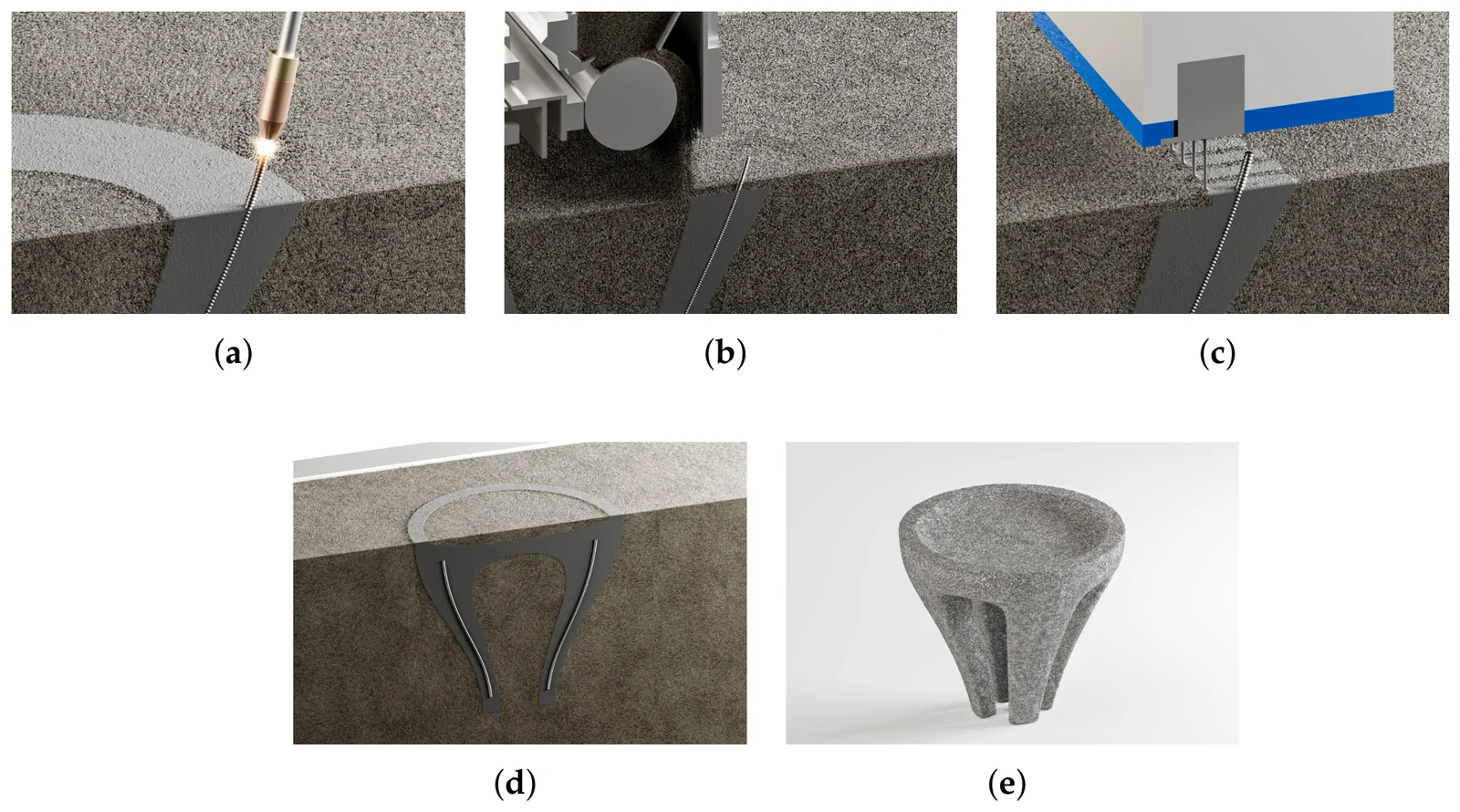
Combined SPI and WAAM process, modified from [3]. (**a**) Printing of a reinforcement bar, (**b**) spreading of an aggregate layer, (**c**) application of cement paste, (**d**) curing of the finished reinforced concrete element, (**e**) excavated component.

Selective Paste Intrusion (SPI) is an additive manufacturing method for producing concrete elements with complex geometries. In the combined SPI and WAAM process, reinforcement bars are printed segment-wise by Wire Arc Additive Manufacturing (WAAM) directly in the growing particle bed of aggregates (Figure 1a). The process then involves sequentially, spreading a layer of aggregates to form a particle bed (Figure 1b), followed by the selective intrusion of cement paste into the voids between the particles (Figure 1c). The unbound surrounding aggregates act as a temporary support structure and enable the fabrication of freeform components without conventional formwork (Figure 1d,e) [1,2].

One research focus in Selective Paste Intrusion is the integration of reinforcement, since this is still a central obstacle for structural applications of concrete additive manufacturing in general, and its interaction with process and form is increasingly treated as a governing design principle [4,5]. Combining Selective Paste Intrusion with WAAM has been identified as a strategy to produce custom-shaped reinforcement structures within the particle bed, and its feasibility has been demonstrated for printed specimens with integrated WAAM bars [6]. Initial investigations have shown that WAAM reinforcements, when tested in conventionally cast concrete, reach bond strengths comparable to reinforcing steel of grade B500B (characteristic yield strength 500 MPa, between ASTM A615 Grades 60 and 75) [7]. Those tests characterise the WAAM bar in a conventionally cast matrix and leave open how the bond forms when the bar is embedded during printing.

The high temperatures generated during the WAAM process can alter the rheological properties of the cement paste. The yield stress and plastic viscosity of cement paste change with temperature, with the largest changes reported below 20 °C and nearly constant values between 30 °C and 40 °C [8]. For cement-based suspensions, temperature alters the apparent viscosity over the range 20 to 80 °C [9], and the coupled effects of time and temperature in superplasticised pastes have been quantified for representative formulations [10].

For the SPI system used here, the relevant thermal envelope was established in a sequence of preceding studies. The fresh-state limit was identified by measuring how rising paste temperatures affect yield stress and viscosity: paste penetration into the particle bed remains sufficient up to 60 °C and deteriorates within a transition range of 60 to 70 °C [11]. The hardened-state limit was confirmed by examining the effect of fresh-state thermal exposure on the compressive and flexural strength of the resulting concrete, which showed no reduction (rather a slight increase) up to 70 °C, with degradation setting in between 70 °C and 80 °C [12]. The effect of elevated temperatures on bond behaviour was investigated separately by pull-out tests in which WAAM reinforcement bars embedded in fresh concrete were heated to 60 °C, 80 °C, and 200 °C immediately after casting, with unheated specimens at 20 °C as reference: moderate exposure (60 to 80 °C) led to a slight reduction in maximum bond strength, while substantial degradation occurred at 200 °C [13]. The actual temperatures occurring during WAAM at varying distances from the welding point were then quantified, and corresponding cooling strategies were evaluated [14]. One such strategy is increasing the vertical distance between the welding point and the particle bed, which reduces the thermal load on the bed. A subsequent study recommended a maximum nozzle-to-bed distance of 50 mm as a balance between mechanical properties and print quality: shape accuracy remained consistent up to 40 mm, whereas at 50 mm the compressive strength declined by about 16% [3]. A vertical protrusion of 40 mm was therefore adopted rather than the recommended maximum of 50 mm: 40 mm is the largest distance at which neither shape accuracy nor compressive strength was impaired, whereas 50 mm accepts a compressive-strength reduction of about 16% in exchange for a larger thermal margin [3]. For the present study, which isolates the geometric shadowing component, the value without strength penalty was chosen so that the bond results are not confounded by a reduced matrix strength. In practice, the choice between 40 mm and 50 mm follows from the thermal load of the selected WAAM parameters [14]: the smaller protrusion is preferable whenever the bed-surface temperature stays below the 70 °C limit [12], and the larger one only where this limit would otherwise be exceeded. The shadowing effects investigated in the present study arise as a direct consequence of this 40 mm process limit. To isolate the geometric component, thermal effects are not considered in the present experimental programme.

**Figure 2:**
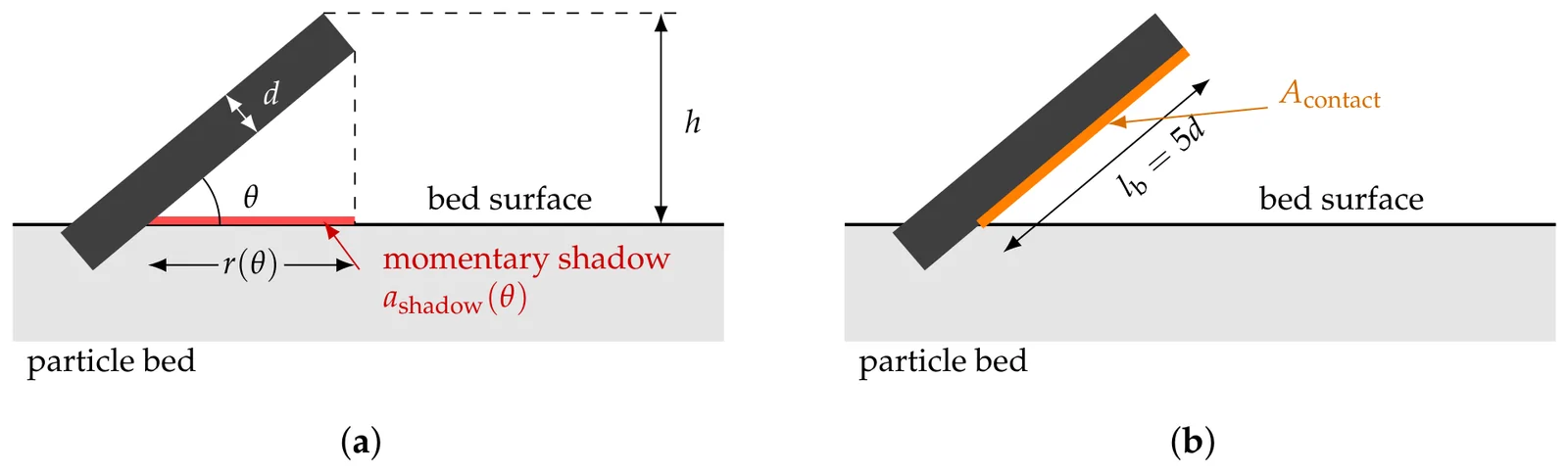
Schematic of the two shadowing manifestations (cross-section, not to scale). (**a**) Projected shadow: while the bar protrudes by up to *h* above the current bed surface, material applied from above cannot reach the strip of the bed surface with horizontal reach r(θ) beneath the bar (red, momentary shadowed area $a_{\mathrm{shadow}}\left(\theta \right)=d\;\cdot \;r\left(\theta \right)$). The union of these momentary shadows over the printing sequence forms the projected shadowed area $A_{\mathrm{shadow}}$ (Section 2.4). The projected shadow grows with bar diameter *d* and with decreasing inclination angle θ, and vanishes at θ=90°. (**b**) Contact zone: the lower half of the bar surface within the bond length $l_{b}$ (orange, $A_{\mathrm{contact}}$) experiences repeatedly disturbed material deposition during overprinting, independent of θ for 0°≤θ<90°.

A protruding reinforcement bar may influence the local material deposition during layer application and overprinting. An elevated bar partially obstructs the transport of aggregates and cement paste into the region beneath it, leading to so-called shadowing effects, see Figure 2. The result is a local inhomogeneity in the concrete matrix and an impaired interface quality between reinforcement and surrounding material, both of which can affect bond behaviour adversely.

The term “shadowing” has previously been used for related deposition-shielding phenomena around reinforcement in concrete additive manufacturing [15,16]. In this study, “shadowing” is defined as a process-induced disturbance of material deposition in the vicinity of a protruding reinforcement bar. Two related manifestations are considered, which can act simultaneously as degradation mechanisms.

The first is a geometrically projected shadowed region in the particle bed. As the bar protrudes above the bed, aggregates and paste applied from above cannot reach the volume that lies in the geometric projection beneath the bar. This region depends on bar diameter and inclination and is most pronounced for larger diameters and shallow inclinations.

The second is a layer-wise modification of the contact zone along the bar surface in the overprinted region. As the structure is built up layer by layer from above, the protruding part of the bar lies above the most recently deposited material at every step of the printing sequence. This generates a zone along the lower half of the bar surface in which material deposition is repeatedly disturbed. Over the printing sequence, this disturbance extends along the full bond length and is largely independent of inclination (Section 2.4).

The mechanistic plausibility of both manifestations rests on two groups of previous results, which differ in how directly they transfer to the SPI conditions used here. The first group was obtained with the same SPI system: the penetration model in [17] was calibrated for quartz sand fractions that include the 1.0 to 2.2 mm fraction used here and related the intrusion depth of the paste to the porosity and grain size of the packing, and the shape-accuracy and strength limits of the nozzle-to-bed distance were established for this system [3]. The second group consists of analogues from granular physics and from other concrete printing processes, which transfer the direction of an effect but not its magnitude: the wall effect at cylindrical boundaries, granular flow past a cylinder, and air-void formation around bars in extrusion-based printing. Penetration models for cement pastes into sand packings show that the intrusion depth depends on the pore structure of the packing, in particular its porosity and grain size [1,17,18], so that a region in the particle bed shielded from aggregate and paste deposition will exhibit a structurally distinct matrix. For the contact zone along the bar surface, the local packing in granular beds at a cylindrical contact differs from the bulk [19,20]: the porosity increases towards the contact [21,22], so that the lower side of the bar is, on purely geometric grounds and prior to any deposition-process consideration, a zone of altered packing. Whether the resulting void volume is later compensated by paste intrusion or remains partially unfilled depends on the local intrusion conditions [17,18]. Either case alters the steel-to-concrete contact compared to a conventionally cast geometry.

Bond stresses are transferred along the interface between reinforcement and surrounding concrete and are commonly expressed per unit of bar surface area within the bond length. The bond behaviour is governed by adhesion, mechanical interlock at the ribs, and friction due to relative displacement [23,24,25]. At the onset of loading, adhesion and local contact mechanisms dominate, but they are lost at very small relative displacements. The bond is then primarily governed by mechanical interlock at the ribs, the dominant load-transfer mechanism over a wide displacement range. With increasing load, damage accumulates and the concrete between the ribs is progressively sheared off, leading to the maximum bond stress. Once this resistance is exhausted, friction along the sheared concrete surface remains as the only relevant load-transfer mechanism. Disturbances in material distribution near the reinforcement may therefore affect both early bond mobilisation and the maximum achievable bond capacity. In addition, sufficient concrete cover or radial confinement is required to ensure that the measured response is governed by pull-out rather than splitting failure [26].

Against this background, three questions are open. The first is whether the vertical protrusion of 40 mm adopted for thermal reasons reduces the bond of the protruding bar through the deposition disturbances described above. The second is how such a reduction scales with bar diameter and inclination angle. The third is which of the two manifestations governs the bond response when both act simultaneously for 0°≤θ<90° and only the projected shadow varies with θ. The present study addresses these questions with a push-through programme of 21 configurations at constant protrusion.

The primary influencing parameters are the bar diameter *d* and the bar orientation relative to the particle bed surface, described by the inclination angle θ between the bar axis and the bed surface (θ=0° for a bar parallel to the bed surface and θ=90° for a bar orthogonal to it, see Figure 2). As the diameter increases, both the projected shadowed region and the layer-wise affected contact zone along the bar surface enlarge. As the inclination angle decreases from 90° towards 0°, the projected shadowed area increases, while the layer-wise contact-zone effect is expected to remain largely unchanged. Two testable hypotheses follow, one for each parameter. Hypothesis H1 (diameter): at every inclination angle, the bond parameters decrease with increasing bar diameter, because both manifestations enlarge with *d*. Hypothesis H2 (angle): at constant diameter, the bond parameters decrease with decreasing inclination angle, because the projected shadowed area grows as θ decreases while the contact-zone manifestation remains unchanged. H2 thus tests specifically whether the projected shadow contributes to the bond response. A confirmation of H1 together with a rejection of H2 would point to the contact-zone manifestation as the governing mechanism.

## 2. Materials and Methods

### 2.1. Objective and Experimental Design

The investigation is based on a systematic test series with varying bar diameters and inclination angles. Bond behaviour was tested in a push-through configuration that adopts the bond length, the loading rate, and the bond stress evaluation from RILEM RC6 [27]. In contrast to the pull-out setup defined there, the bar was loaded in compression (Section 2.3). RILEM RC6 formulates its procedure for bar diameters of at least 10 mm and for comparisons between bars of approximately equal diameter. The present series extends the configuration to 8 mm and to comparisons across diameters, so the RC6-derived values are internally consistent comparative measures rather than normative bond strengths.

### 2.2. Materials

Specimens were produced using the Selective Paste Intrusion process. Aggregate layers with a thickness of 3 mm were deposited successively and selectively infiltrated with cement paste until the target height was reached.

Quartz sand with a grain size of 1.0 to 2.2 mm was used as aggregate ($d_{50}=1.6$ mm, particle density 2643 kg $m^{-3}$, bulk density 1447 kg $m^{-3}$, packing porosity 0.453, determined for the same sand type from an earlier delivery [17]). With the complete filling of the pore space, the paste content of the printed concrete corresponds to the bed porosity, about 42% by volume. The cement paste consisted of an ordinary Portland cement with strength class 42.5 (CEM I 42.5 N per EN 197-1 [28], Heidelberg Materials, Heidelberg, Germany, density about 3.10 kg ${dm}^{-3}$) with a water-to-cement ratio of 0.40 and a polycarboxylate-ether-based superplasticiser (MasterCO_2_re 3240, Master Builders Solutions Deutschland GmbH, Trostberg, Germany) at 0.8% by weight of cement. The mix proportions correspond to those used in [13] (580 kg $m^{-3}$ cement, 232 kg $m^{-3}$ water, and 1537 kg $m^{-3}$ sand per cubic metre of concrete), which gives a calculated paste density of about 1.93 kg ${dm}^{-3}$. The paste was mixed at 20 °C (±0.5 °C) and applied in a laboratory at 20 °C. The flowability of the cement paste was characterised by its spread-flow, measured with a Haegermann cone (cone geometry as specified in EN 1015-3 [29]) placed on a flat glass plate and lifted vertically. In contrast to the jolting procedure of the standard, the paste was allowed to spread under gravity without any jolts, so that the spread is governed by the paste yield stress alone. The spread was adjusted to 400 to 410 mm by varying the superplasticiser dosage. For an SPI concrete with the same aggregate and the same spread-flow level (400 mm), produced with a paste of water-to-cement ratio 0.30, no dependence of the compressive strength on the loading direction relative to the layers was observed [2]. Whether this transfers to the water-to-cement ratio of 0.40 used here is examined in Section 3, together with the angle dependence of the bond data.

Ribbed reinforcing steel bars of grade B500B [30] (characteristic yield strength $f_{\mathrm{yk}}=500\;\mathrm{MPa}$, ductility class B) were used as reinforcement. Their diameters, geometry, and configuration are described in Section 2.3.

After 24 h, the specimens were excavated from the particle bed and subsequently stored for at least 28 days under constant ambient conditions (20 °C, 65% relative humidity) prior to testing.

### 2.3. Specimen Geometry and Reinforcement Configuration

Ribbed reinforcing bars of grade B500B with diameters of 8 mm, 16 mm, and 25 mm were used. The bars were embedded at inclination angles θ between 0° (parallel to the particle bed surface) and 90° (orthogonal to the particle bed surface) in 15° increments. Five specimens were produced for each combination of diameter and angle.

The maximum protrusion of the reinforcement was limited to 40 mm above the particle bed surface, measured vertically, corresponding to the largest nozzle-to-bed distance at which shape accuracy remained consistent in [3]. The protrusion was kept constant for all configurations, so all results refer to this single boundary condition (Section 4).

**Figure 3:**
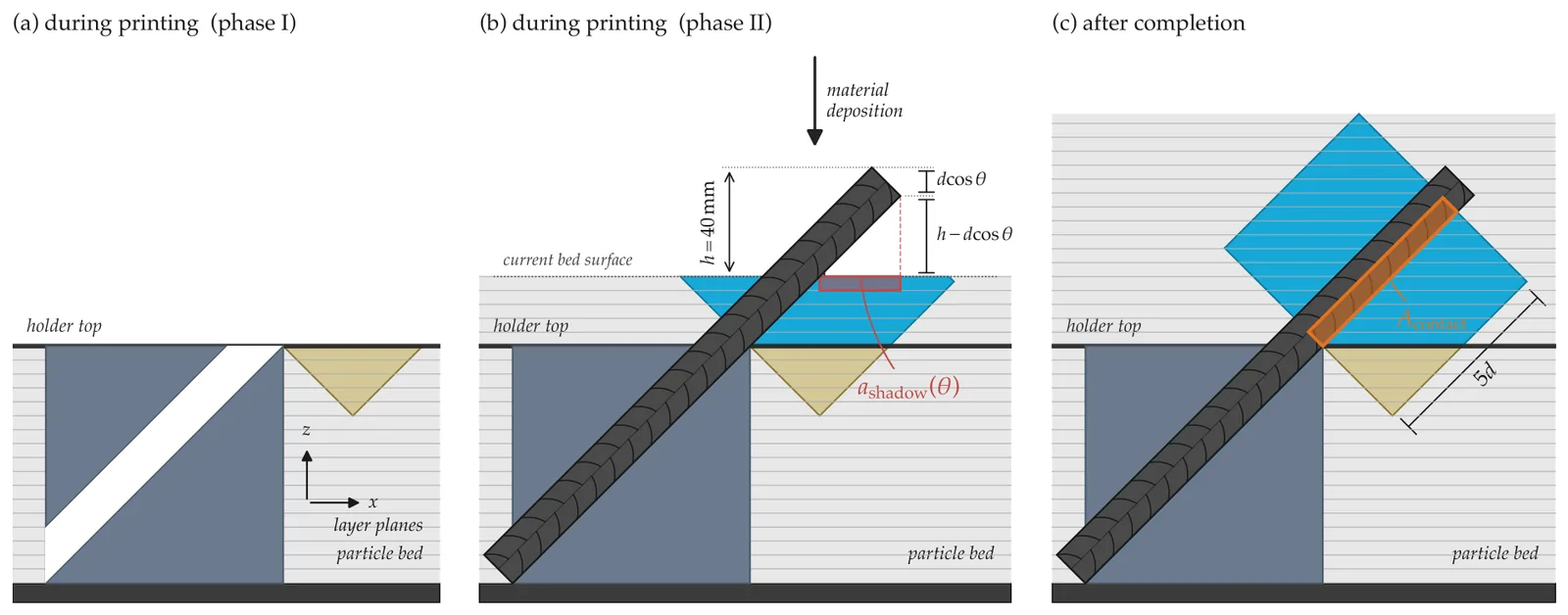
Cross-sectional schematic of the specimen geometry and the two shadowing manifestations in the vertical plane containing the bar axis (*x* horizontal, *z* build direction, layers horizontal). (**a**) Printing phase I: the lower specimen portion (yellow) is produced without the bar, and the particle bed is filled up to the holder top. (**b**) Printing phase II, the overprinting with the bar in place (blue area): the bar protrudes by up to h=40 mm above the current bed surface, and the momentary shadow (red outline, $a_{\mathrm{shadow}}\left(\theta \right)$) marks the geometrically shielded region. The shadowed area itself lies on the bed surface. The red patch marks its trace in the cross-sectional plane. The union of these momentary shadows over the printing sequence forms $A_{\mathrm{shadow}}$. (**c**) Completed specimen: the holder (slate-grey) supports the bar, and $A_{\mathrm{contact}}$ (orange) marks the affected contact zone along the lower half of the bar surface within the bond length 5d.

According to RILEM RC6 [27], a bond length of 5d is defined, corresponding to 40 mm, 80 mm, and 125 mm for bar diameters of 8 mm, 16 mm, and 25 mm, respectively. With a constant vertical protrusion of h=40 mm, measured to the upper tip of the bar, the required bond length exceeds the available bar length above the particle bed for most diameter–angle combinations. The bars must therefore be segmented and integrated stepwise (Section 2.5). The maximum bar length per segment follows from the condition that the upper tip of a protruding segment must not exceed the protrusion limit *h*. The vertical extent of a protruding segment consists of the axial rise $L_{\mathrm{eff}}\sin\theta$ and the vertical width dcosθ of the inclined bar cross-section, measured between the end of the lower bar surface and the upper tip. Setting $L_{\mathrm{eff}}\sin\theta +d\cos\theta =h$ and solving for the segment length gives $L_{\mathrm{eff}}=\left(h-d\cos\theta \right)/\sin\theta$ for θ>0° (all lengths in mm). In descriptive terms, the correction dcosθ reflects that the bar is not an infinitely thin axis: the inclined cross-section itself occupies part of the admissible protrusion height (see the dimension chain in Figure 3b). As a consequence, $L_{\mathrm{eff}}$ does not vary monotonically with the inclination angle. Starting from 40 mm at θ=90°, it first decreases with decreasing angle, reaches a minimum between 45° and 75° depending on the diameter, and only then increases again, because a flatter bar needs more axial length for the same vertical rise (Table 1). This non-monotonic behaviour carries over to the segmentation, since no segment may exceed $L_{\mathrm{eff}}$. The required bar length above the bed is the bond length plus 10 mm (±2 mm) of free bar end for the displacement sensor. For all configurations with more than one segment, the as-built segment lengths were chosen as practical lengths that do not exceed $L_{\mathrm{eff}}$ beyond the cutting tolerance of ±2 mm, and the last segment of each bar was shortened to the remaining length required to complete the bond length plus the free bar end. If $L_{\mathrm{eff}}$ falls below a round length, the segments follow $L_{\mathrm{eff}}$ directly: at 60° and 75° for 16 mm, for example, $L_{\mathrm{eff}}=37.0$ mm and 37.1 mm limit the segments to 37 mm, so the as-built division is 37+37+16. The same rule produces the divisions in the 25 mm series, for example 30+30+30+30+15 at 45°, where $L_{\mathrm{eff}}=31.6$ mm. Table 1 compares the available and required lengths, gives the resulting number of segments, and lists the as-built segment lengths.

All lengths in this derivation and in Table 1 use the nominal bar diameter *d*. The ribs raise the outer profile to $d+2h_{m}$, with the mean rib heights $h_{m}$ reported in Section 3 (0.83 mm, 1.34 mm, and 1.91 mm). Inserting $d+2h_{m}$ in place of *d* reduces $L_{\mathrm{eff}}$ by up to 5% for 8 mm, 11% for 16 mm, and 23% for 25 mm, with the largest reduction at 15° (61.2 mm to 47.0 mm). Since the as-built segments follow the nominal $L_{\mathrm{eff}}$, the rib crests of a freshly placed segment momentarily protrude beyond the 40 mm limit by $2h_{m}\cos\theta$ plus the excess of the segment length over the nominal $L_{\mathrm{eff}}$, at most 3.4 mm (25 mm at 15°) and below 2 mm for all 8 mm configurations, in addition to the cutting tolerance of ±2 mm, until the next layers reduce the protrusion. The ribbed profile enlarges the momentary reach in Equation (2) and scales the projected shadowed area of Equation (3) by ${\left(\left(d+2h_{m}\right)/d\right)}^{2}$, a factor of 1.46, 1.36, and 1.33 for the three diameters, which leaves the ordering of $A_{\mathrm{shadow}}$ between the configurations unchanged. The ribs also act as local obstacles to particle transport at the scale of the rib height, an effect that the descriptors do not resolve and that is discussed with the rib geometry in Section 3.

For the special case of 0°, the bar was positioned entirely above the particle bed at a constant vertical offset of 40 mm, measured to the upper bar edge. In this configuration, the bar was longer than the bond length and extended beyond the printed specimen on both sides. Only the bond length was overprinted, while the protruding bar ends on both sides served for load application and displacement measurement.

The need for segmentation has implications for the choice of test method. The individual bar segments were joined with a thin layer of cyanoacrylate adhesive during specimen fabrication. The adhesive provided only temporary fixation and did not contribute to load transfer during testing. In a conventional pull-out test, tensile forces would act on the adhesive joints at the segment interfaces and could lead to premature failure at these locations rather than mobilising the bond between reinforcement and concrete. To avoid this, a modified push-through test was performed, in which the bar is pushed through the specimen rather than pulled. This results in purely compressive loading of the reassembled segmented bar, so that the adhesive joints do not lead to premature failure. The measured force–displacement behaviour thus reflects the steel-to-concrete bond rather than the strength of the segment joints. Local irregularities of the bond in the immediate vicinity of the joints may nonetheless remain and constitute a process-related source of scatter. A continuous bar can be placed only where the required length does not exceed $L_{\mathrm{eff}}$, which is the case for the continuous configurations of Table 1. For all other configurations, the protrusion limit itself enforces the segmentation, so a control series with continuous bars of the same geometry cannot be produced in the particle bed. The programme therefore contains a partial control rather than a dedicated one: the 8 mm series comprises continuous bars from 0° to 45° and segmented bars from 60° to 90°, and the number of segments varies from one to five across the configurations. This comparison is evaluated in Section 3.

### 2.4. Bond Stress and Geometric Shadowing Descriptors

The bond stress τ was calculated assuming a uniform bond stress over the bond length, following the evaluation formula of RILEM RC6 [27] without its normalisation to a reference concrete strength, which is not required here because all specimens were produced with the same concrete, as

(1) $$\tau =\frac{F}{\pi \;\cdot \;d\;\cdot \;l_{b}}$$

with *F* the recorded force in N, *d* the bar diameter in mm, and $l_{b}=5d$ the bond length in mm, so that τ follows in MPa.

To quantify the geometric component of shadowing, the momentary shadow cast by the protruding bar is considered first. At any stage in the printing sequence, a bar segment protrudes above the current bed surface by up to h=40 mm, measured vertically to the upper tip of the bar (Figure 3b). Material applied vertically from above is largely prevented from reaching the strip of the bed surface that lies in the horizontal projection of the protruding part. For a bar of diameter *d* at inclination angle θ, this momentary shadow extends from the point at which the lower bar surface exits the bed. Its horizontal extent, the reach r(θ) in mm, is

(2) $$r\left(\theta \right)=\frac{h-d\cos\theta }{\tan\theta }$$

valid for 0°<θ<90°, where the term dcosθ accounts for the vertical offset between the upper bar tip, at which the protrusion is measured, and the end of the lower bar surface, from which the shadow extends (*h* and *d* in mm). The two limiting angles are treated below. Both contributions are indicated by the dimension chain in Figure 3b. The momentary shadowed area on the bed surface is $a_{\mathrm{shadow}}\left(\theta \right)=d\;\cdot \;r\left(\theta \right)$, in mm^2^.

The momentary shadow is not stationary. As printing proceeds, the bed surface rises layer by layer, the protrusion of the current segment decreases, and the shadow shortens while its starting point travels up the bar. Once a segment is fully overprinted, the next segment restores the protrusion and the same process repeats. Each fully overprinted segment of length $\ell_{i}$ along the bar axis, with the running index $i=1,\ldots ,n_{s}$ numbering the segments in Table 1, advances the starting point of the shadow by its horizontal projection $\ell_{i}\cos\theta$. The union of all momentary shadows over the printing sequence therefore forms a continuous strip on the bed surface beneath the bar within the concrete specimen. The accumulated shadowed area follows in three steps: the strip contributions of the $n_{s}$ segments are summed, the segment lengths within the bond length add up to the bond length ($\sum_{i=1}^{n_{s}}\ell_{i}=l_{b}$, with $n_{s}=1$ for the continuous configurations of Table 1), and the RILEM bond length $l_{b}=5d$ is inserted:

(3) $$A_{\mathrm{shadow}}=d\;\cdot \;\sum_{i=1}^{n_{s}}\ell_{i}\cos\theta =d\;\cdot \;l_{b}\;\cdot \;\cos\theta =5\;d^{2}\cos\theta ,\;0{}^{\circ}\leq \theta \leq 90{}^{\circ}$$

with *d*, $\ell_{i}$, and $l_{b}$ in mm and $A_{\mathrm{shadow}}$ in mm^2^. The result is independent of the number and lengths of the individual segments and of the protrusion height *h*, which enter only the momentary shadow. The finite-diameter correction dcosθ in Equation (2) shifts only the front of the momentary shadow within the strip and does not alter the total swept area. Equation (3) thus depends only on *d* and θ, not on the fabrication parameters *h* or the segmentation. This distinguishes $A_{\mathrm{shadow}}$ from $A_{\mathrm{contact}}$ (introduced below), which is independent of θ. For θ=0° (bar parallel to the particle bed), no segmentation occurs and the shadow is no longer described by Equation (2): the entire lower side of the bar shades the bed simultaneously, bounded by the overprinted bond length. In this limiting case, the momentary shadow spans the entire strip at once, $a_{\mathrm{shadow}}$ and $A_{\mathrm{shadow}}$ coincide, and Equation (3) reduces to $d\;\cdot \;l_{b}$. For all inclined configurations, the momentary shadow is a shorter strip that travels along the bar during overprinting. For θ=90° (bar orthogonal to the particle bed), the projected shadow vanishes.

To describe the layer-wise process-induced manifestation of shadowing, the affected region is taken to be the lower half of the bar surface within the bond length:

(4) $$A_{\mathrm{contact}}=\frac{\pi \;\cdot \;d\;\cdot \;l_{b}}{2}=\frac{5}{2}\;\pi \;d^{2},\;0{}^{\circ}\leq \theta <90{}^{\circ}$$

where the second expression follows by inserting the RILEM bond length $l_{b}=5d$ (*d* and $l_{b}$ in mm, $A_{\mathrm{contact}}$ in mm^2^). The lower-half definition is a geometric working assumption for the affected region, not a measured extent. It fixes the descriptor to a definite, diameter-scaled fraction of the bar surface so that the hypothesis can be tested comparatively across diameters and angles. The actual extent and intensity of the disturbance are not measured in this study (Section 4). The contact-zone mechanism is therefore a hypothesis that the push-through data can test only indirectly, through the diameter and angle dependence of the bond parameters.

The geometric reasoning rests on the sequential overprinting of the protruding bar segments. At every printing step, a portion of the bar protrudes above the particle bed by up to 40 mm, and the material applied from above (aggregate during spreading and cement paste during intrusion) is blocked from the lower side of this protruding portion. The degree to which trickling aggregate and locally flowing cement paste compensate this blockage is addressed in Section 3. Once a segment is fully overprinted, a new segment is added that again protrudes by 40 mm, and the same shielding mechanism repeats. For the special case θ=0°, where the bar lies parallel to the bed and is not segmented, the lower side is shielded continuously rather than segment-wise. The affected region, the lower half of the bar surface, is the same. Over the sequence of segments, the lower half of the bar surface remains continuously shielded and the resulting disturbance extends along the full bond length. Owing to the cylindrical symmetry of the bar, the shielded half always covers half of the lateral surface area, regardless of inclination, as long as a defined upper and lower side of the bar exists. The descriptor is therefore taken as constant in θ for 0°≤θ<90°. At θ=90° the bar axis becomes parallel to the deposition direction, no upper or lower side can be defined, and only a small front-facing cross-section of area $\pi d^{2}/4$ (plus the local rib protrusions) obstructs the material flow. This end face is not part of the bond-transferring bar surface. The mechanism described by $A_{\mathrm{contact}}$ therefore ceases to apply at this angle, and the configuration is treated as a special case in the discussion. The $d^{2}$ form of Equations (3) and (4) is a direct consequence of the RILEM bond length convention $l_{b}=5d$ [27] rather than a mechanistic prediction in itself. Both descriptors are illustrated in Figure 3: the momentary shadow, whose union over the printing sequence forms $A_{\mathrm{shadow}}$, is shown in red on the particle bed surface beneath the protruding bar, and $A_{\mathrm{contact}}$ is shown in orange along the lower half of the bar surface within the bond length.

### 2.5. Specimen Fabrication and Setup

The specimens were inclined cylinders with an as-printed diameter of 100 mm. Their longitudinal axis was aligned with the prescribed bar inclination angle. The reference frame is fixed by the particle bed: the layers are horizontal, the build direction *z* is vertical, and the inclination angle θ is measured between the bar axis and the horizontal bed surface in the vertical plane containing the bar axis (Figure 3). The specimen axis coincides with the bar axis, and the push-through load is applied along this common axis (Section 2.7), so the angle between the loading direction and the layer planes equals θ. The vertical plane of the bar axis was oriented perpendicular to the travel direction of the spreading blade and the paste nozzle. To realise this geometry within the SPI process, a cubic holder with an edge length of 100 mm was placed on the base plate of the SPI printer. The holder contained a through-hole whose axis followed the prescribed inclination angle and whose diameter matched the bar diameter. Figure 3 shows a cross-sectional schematic of the specimen geometry and the fabrication process.

Printing proceeded in two phases. In phase I, the lower part of the specimen (yellow area) was printed without the bar. Because of the inclination, this part lay below the holder top and therefore had to be produced before the bar was inserted. Before printing, the upper opening of the hole was sealed with adhesive tape to prevent aggregate from entering during the first printing phase.

After phase I, the adhesive tape was removed and the reinforcement bar was inserted through the hole in the holder. In phase II, printing continued with the bar in place (blue area). The print head was kept at a constant offset of 40 mm above the current bed surface [3], so that the bar initially protruded by this amount above the bed surface. With each successive layer, the vertical protrusion decreased by the layer thickness until the lower edge of the bar at its tip reached the level of the bed surface. At this stage, the full bar cross-section was exposed and the next bar segment was butt-joined with cyanoacrylate adhesive (Loxeal 32 Ethyl, Cesano Maderno, Italy), restoring the original protrusion of 40 mm to the upper tip, and printing continued. This procedure was repeated until the upper part of the specimen reached the height corresponding to the bond length above the holder top. The length of the final segment was chosen such that it did not exceed 40 mm and that 10 mm (±2 mm) of free bar remained above the printed specimen for mounting the displacement sensor. For configurations at 15° to 90°, the portion of the bar inside the holder constituted the unbonded length on the loading side during the subsequent push-through test.

To minimise geometric irregularities at the segment joints, the segments were prepared in advance by cutting a continuous bar with thin cutting blades and were then reassembled in their original order and orientation.

**Figure 4:**
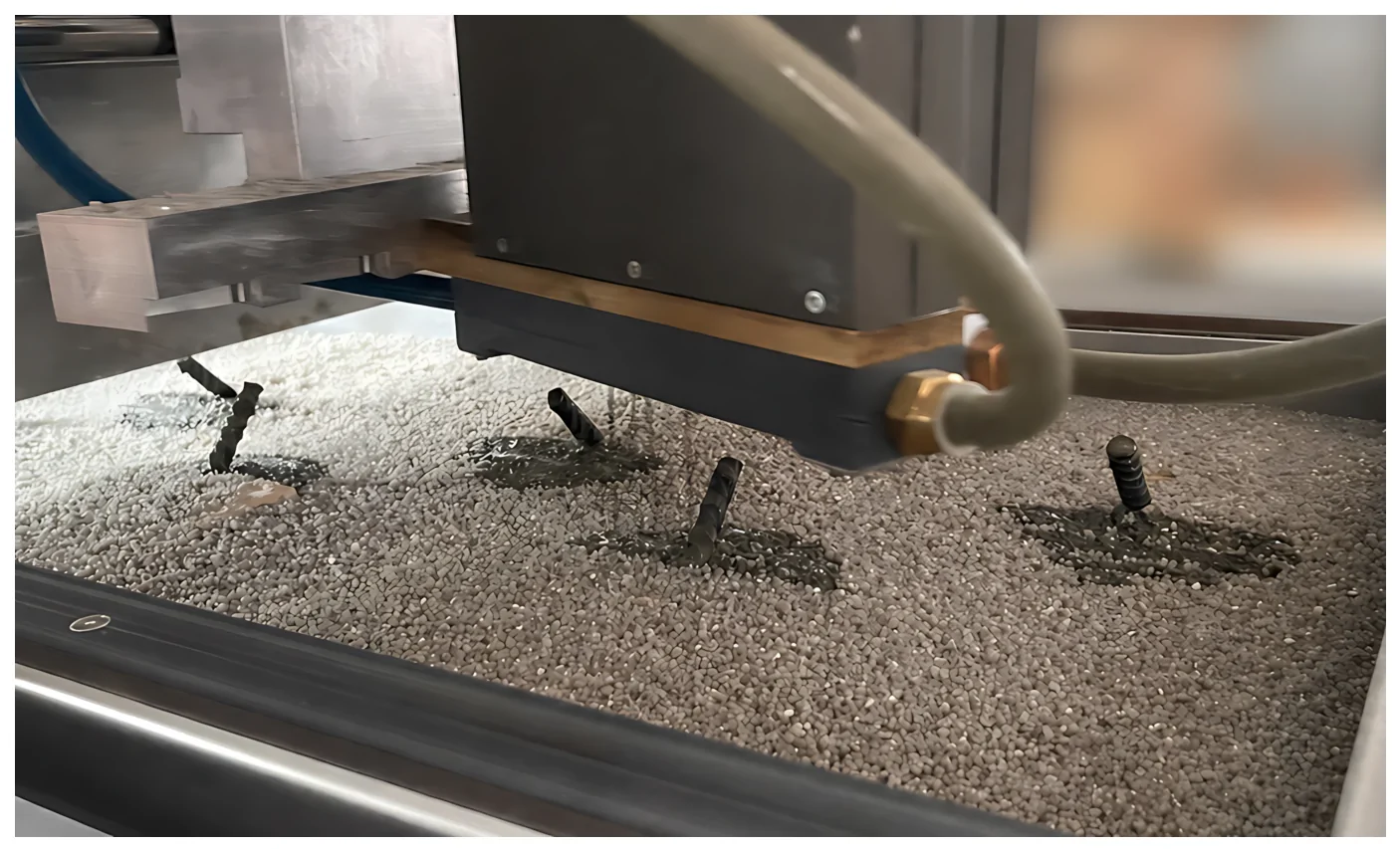
Configuration during the SPI printing process with protruding reinforcement bars.

Figure 4 shows the configuration during printing.

### 2.6. Post-Processing and Specimen Preparation

To prevent splitting failure across the diameter range, the specimens were inserted into pre-cut steel confinement rings. For this purpose, all specimens were first reduced from their initial diameter of 100 mm to a uniform outer diameter of 80 mm by wet core drilling with a diamond core bit along the bar axis, which ensured a defined specimen geometry and a uniform lateral surface for bonding to the ring. The drilled surface lies at least 27.5 mm (25 mm bars) to 36 mm (8 mm bars) from the bar surface, so the bond zone is not intersected by the cut. The rings were dimensioned with a height equal to the bond length 5d, so that the height of the embedded section after trimming corresponds exactly to the bond length. The steel rings were sandblasted prior to bonding to improve adhesion and bonded using an epoxy adhesive (Hilti HIT-RE 500, Schaan, Liechtenstein). The combined effect of the residual concrete cover and the steel ring (steel tube 88.9 mm outer diameter, wall thickness 3.2 mm) was dimensioned to provide radial confinement at all bar diameters, so that the measured bond response is not dominated by splitting. No splitting cracks were observed on the specimen faces after testing.

After adhesive curing for 48 h at 20 °C and 65% relative humidity, excess concrete above and below the rings was removed using a fine tile saw, so that the resulting specimens have a height equal to the defined bond length. The saw cuts intersect the bar at the two end faces of the bond length. Possible local damage at these faces affects the outermost millimetres of the bond length equally for all configurations and enters the evaluation through the nominal bond length 5d in Equation (1), so it can lower the absolute bond stresses slightly but does not bias the comparison between configurations. The free bar end on the loading side was not cut (Section 2.7).

### 2.7. Mechanical Testing

**Figure 5:**
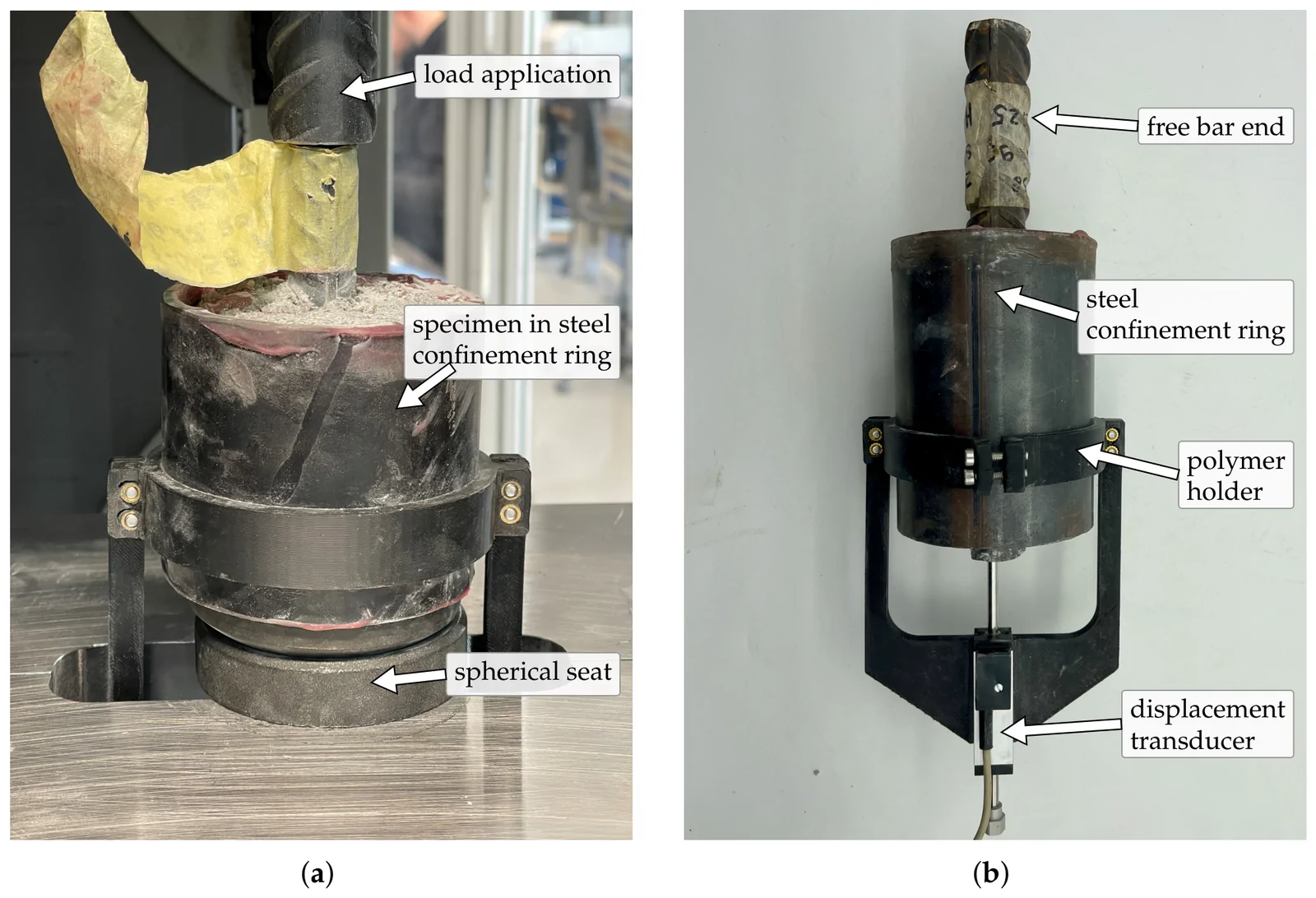
Push-through test configuration. (**a**) Specimen placed on the spherical seat in the testing machine, with the reinforcement bar protruding upward for load application. (**b**) Specimen with the polymer holder clamped to the steel confinement ring, providing the mounting point for the linear displacement transducer at the free bar end.

Bond behaviour was determined using a modified push-through test on a universal testing machine (Zwick Z600, Ulm, Germany), as shown in Figure 5a. The specimen was placed on a spherical seat to compensate for minor angular deviations introduced during core drilling and to ensure axial load application along the bar axis.

The bar protruded beyond the bonded section on the loading side. This protruding length was deliberately not trimmed after specimen preparation so as not to disturb the bond zone at the specimen face.

The test was run in force-controlled mode. The loading rate was adopted from RILEM RC6 [27] and computed as $\dot{F}=0.5\;\cdot \;d^{2}$, with the bar diameter *d* inserted numerically in mm and the resulting loading rate in $N\;s^{-1}$. This gives nominal loading rates of 32 $N\;s^{-1}$, 128 $N\;s^{-1}$, and 312.5 $N\;s^{-1}$ for the 8 mm, 16 mm, and 25 mm bars, respectively.

Slip was measured at the unloaded bar end (the free end opposite to the load application point) using a linear displacement transducer (Ahlborn FWA025TR, Holzkirchen, Germany) mounted in a polymer holder clamped to the steel confinement ring (Figure 5b). Force was recorded using the internal load cell of the testing machine.

### 2.8. Data Evaluation

For each diameter–angle combination, five replicate specimens were produced, except for 8 mm at 75°, where four were tested, and between three and five entered the evaluation. The number of evaluated specimens per configuration is given with the results in Section 3. Seven tests were excluded for one of two reasons. (i) No usable slip signal was obtained during the load rise because the displacement transducer failed, and the test was stopped (three tests of the 8 mm series (two at 30°, one at 90°), and one test each of the 16 mm and 25 mm series at 90°). (ii) The displacement signal showed an offset at the start of loading, so that the slip levels could not be assigned, whereas the force record is complete (one test for each of the 16 mm series at 90° and of the 25 mm series at 60°). The excluded tests, their recorded forces, and the position of these forces relative to the group means are listed in Table A1. The bond stresses calculated from the recorded forces lie between 29% below and 58% above the means of their groups (four above, three below), so the exclusions do not shift the groups in one direction. For the five tests that met criterion (i), the record ends at the stop during the load rise, so their forces are not maximum forces. Only the two tests that met criterion (ii) reached a force maximum. Including them would raise the group means at $\tau_{\max}$ (the maximum bond stress, defined below) from 17.7 MPa to 18.7 MPa (16 mm, 90°) and from 15.1 MPa to 16.8 MPa (25 mm, 60°), which does not alter the diameter ordering described in Section 3. Bond stresses were extracted at four characteristic slip levels, $\tau_{0.001}$, $\tau_{0.01}$, $\tau_{0.1}$, and $\tau_{\max}$. The numeric subscript denotes the slip in mm at which the bond stress is evaluated: $\tau_{0.001}$ is read at a slip of 0.001 mm (1 μm), $\tau_{0.01}$ at 0.01 mm, and $\tau_{0.1}$ at 0.1 mm, while $\tau_{\max}$ is the maximum bond stress, taken at the slip $s(\tau_{\max})$ at which it occurs. These four levels capture the response at very small slip, the early interlock stage, the developed interlock stage, and the bond capacity, respectively. At a slip of 1 μm, $\tau_{0.001}$ lies at the resolution limit of the test setup (bedding-in of the spherical seat, compliance of the load path and of the segment joints) and is therefore best read as the system response at very small slip rather than as a pure chemical-adhesion measure.

## 3. Results and Discussion

**Figure 6:**
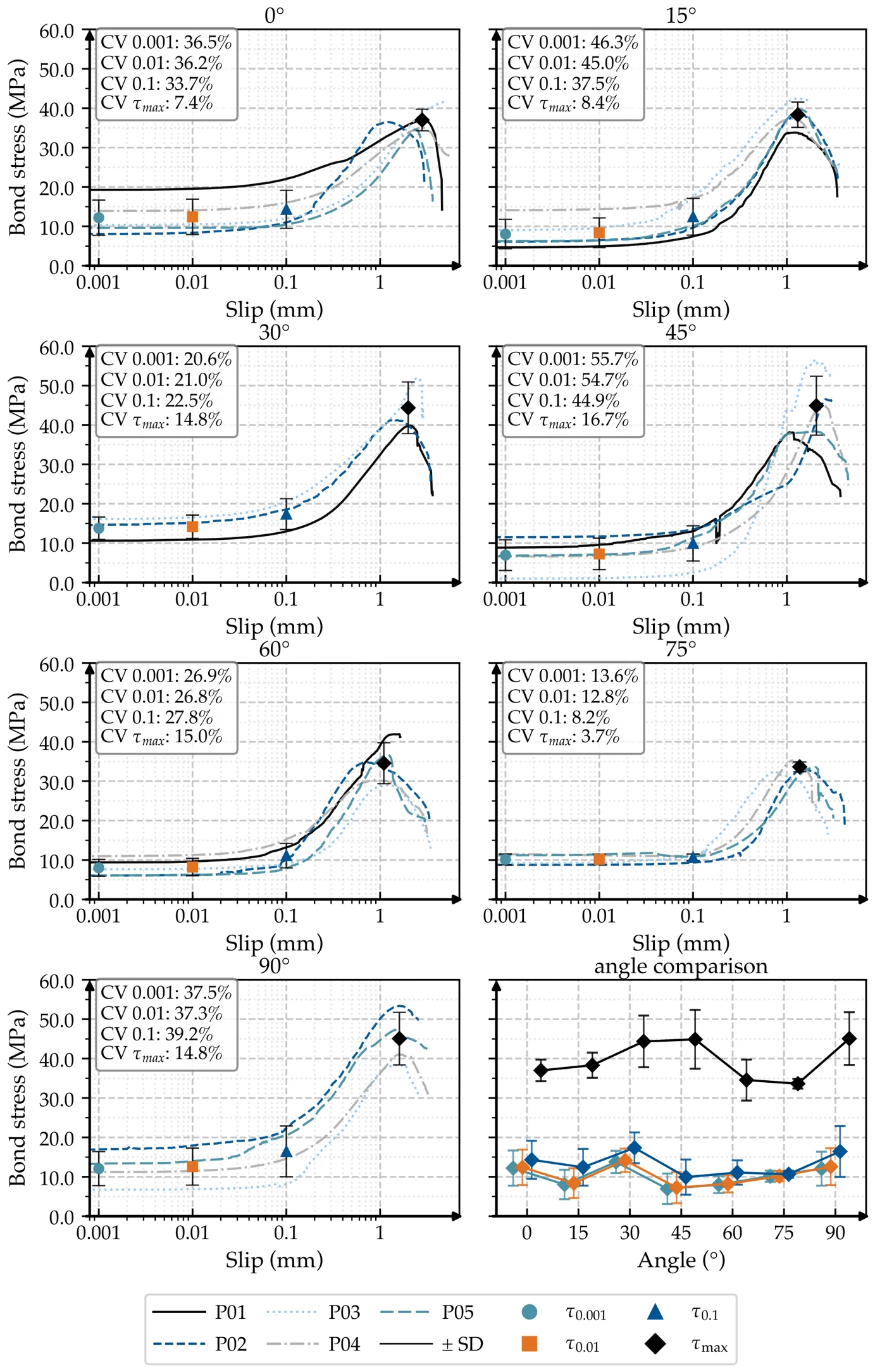
Bond stress–slip curves and characteristic bond values for the 8 mm specimens. The boxes state the coefficients of variation (CV) of the replicates at $\tau_{0.001}$, $\tau_{0.01}$, and $\tau_{0.1}$ and at the maximum bond stress $\tau_{\max}$. The markers for $\tau_{\max}$ are placed at the individual slip $s(\tau_{\max})$ of each curve. Replicates are distinguished by colour and line style, and the four slip levels by marker shape, so that the panels remain readable in greyscale.

**Figure 7:**
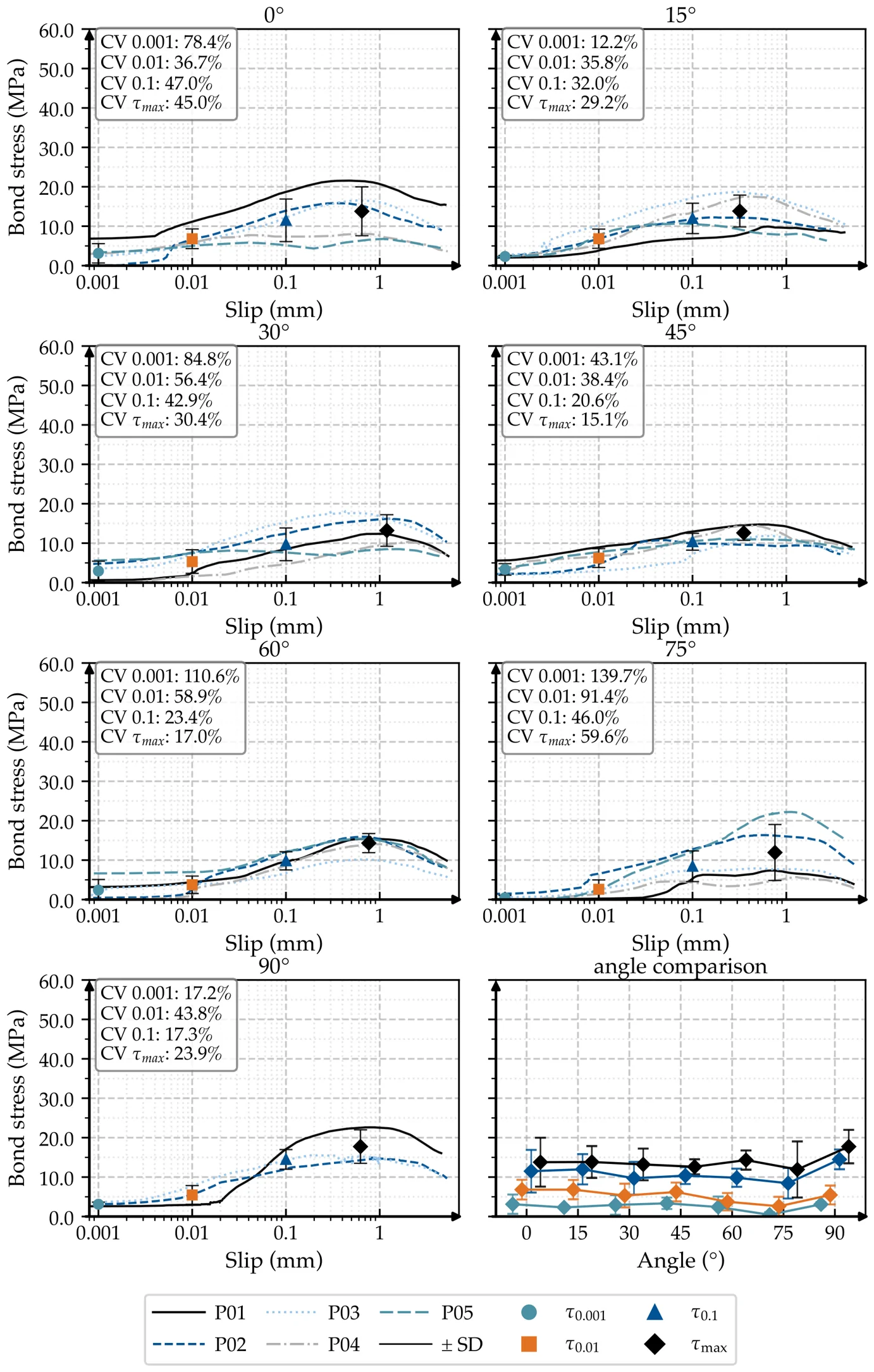
Bond stress–slip curves and characteristic bond values for the 16 mm specimens. The boxes state the coefficients of variation (CV) of the replicates at $\tau_{0.001}$, $\tau_{0.01}$, and $\tau_{0.1}$ and at the maximum bond stress $\tau_{\max}$. The markers for $\tau_{\max}$ are placed at the individual slip $s(\tau_{\max})$ of each curve. Replicates are distinguished by colour and line style, and the four slip levels by marker shape, so that the panels remain readable in greyscale.

**Figure 8:**
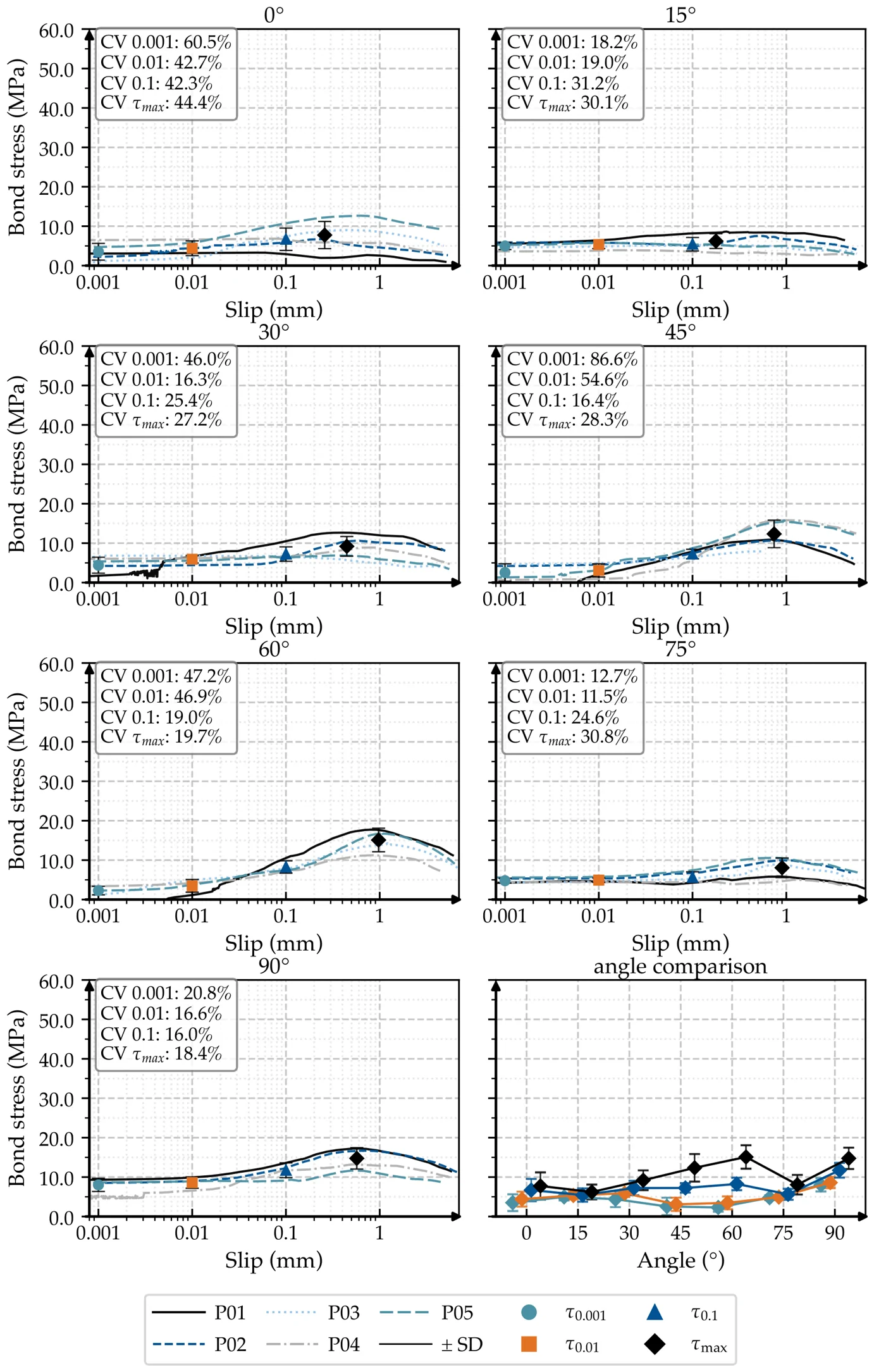
Bond stress–slip curves and characteristic bond values for the 25 mm specimens. The boxes state the coefficients of variation (CV) of the replicates at $\tau_{0.001}$, $\tau_{0.01}$, and $\tau_{0.1}$ and at the maximum bond stress $\tau_{\max}$. The markers for $\tau_{\max}$ are placed at the individual slip $s(\tau_{\max})$ of each curve. Replicates are distinguished by colour and line style, and the four slip levels by marker shape, so that the panels remain readable in greyscale.

The bond stress–slip curves for the three diameters are shown in Figure 6, Figure 7 and Figure 8. Each figure presents the individual curves for all replicates per inclination angle, the mean bond stresses at the four characteristic slip levels, and the angle dependence in the bottom-right panel.

Table 2 summarises the mean bond stresses and standard deviations at the four characteristic slip levels for all diameter–angle combinations.

Across all investigated parameters, the bond stress–slip curves exhibit the same characteristic shape. A shallow initial increase at very small displacements is followed by a steeper rise as mechanical interlock develops, before $\tau_{\max}$ is reached. A subsequent decrease occurs due to progressive damage within the bond zone. This sequence is in line with the classical adhesion–interlock–friction phasing reported for ribbed bars in conventional concrete [23,24].

**Figure 9:**
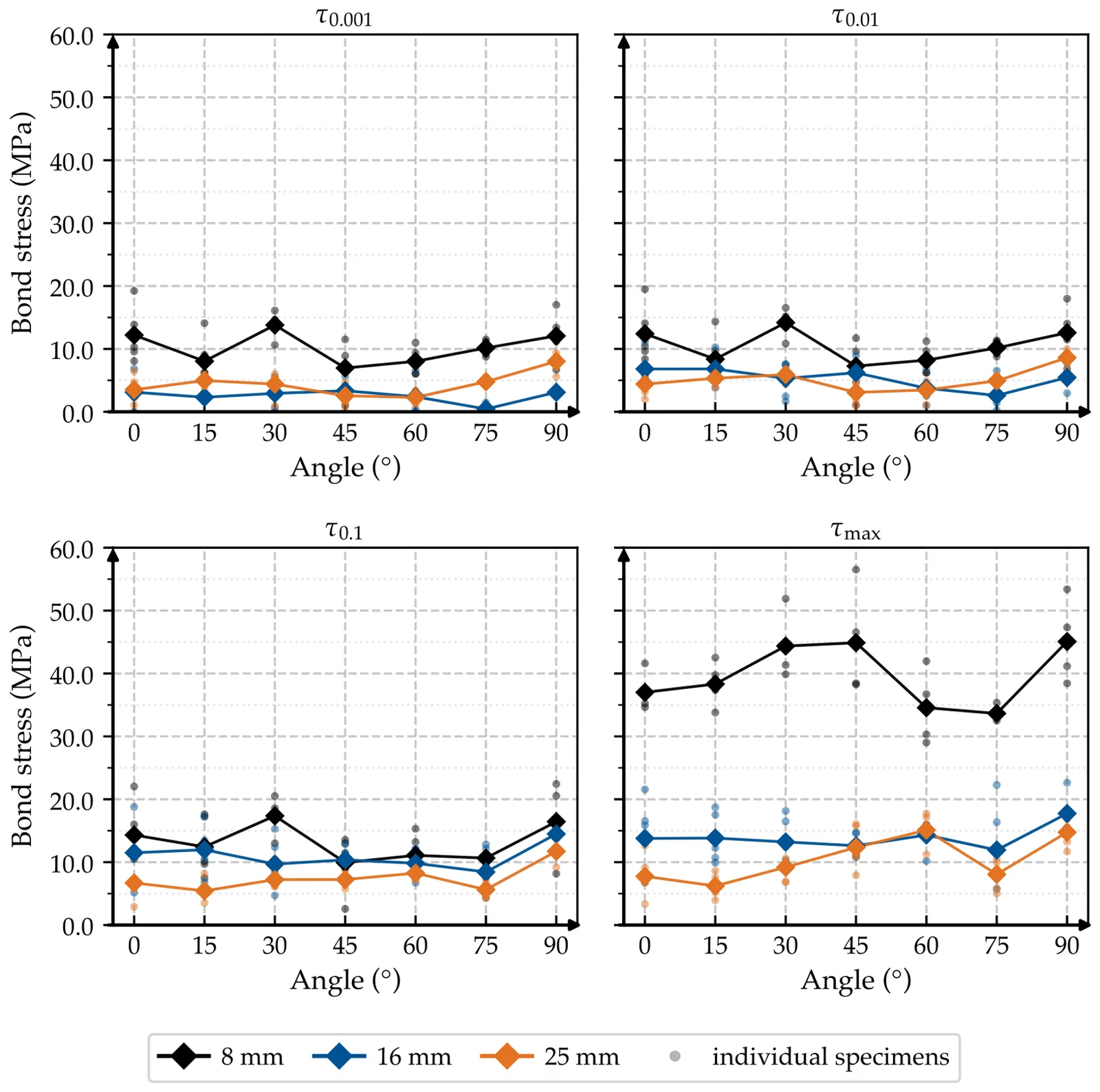
Bond parameters $\tau_{0.001}$, $\tau_{0.01}$, $\tau_{0.1}$, and $\tau_{\max}$ (slip levels in mm, Section 2.8 for definitions) as a function of the inclination angle for the three investigated bar diameters. Lines connect the mean values per inclination angle. The small markers show the individual specimen values.

The mean bond parameters $\tau_{0.001}$, $\tau_{0.01}$, $\tau_{0.1}$, and $\tau_{\max}$ as a function of inclination angle for the three diameters are shown in Figure 9.

The bar diameter dominates the bond response, in line with hypothesis H1. The 8 mm bars reach the highest bond stresses at every angle and every slip level, with one exception at $\tau_{0.1}$ and 45°, where the 16 mm mean (10.4 MPa) exceeds the 8 mm mean (9.9 MPa). At the developed-interlock and capacity levels ($\tau_{0.1}$ and $\tau_{\max}$), the full ordering τ(8 mm)>τ(16 mm)>τ(25 mm) holds in the angle means with two exceptions: at 60° the 25 mm mean exceeds the 16 mm mean at $\tau_{\max}$ (15.1 MPa against 14.3 MPa), and at 45° the 16 mm mean exceeds the 8 mm mean at $\tau_{0.1}$ (10.4 MPa against 9.9 MPa). For $\tau_{\max}$, mean values for the 8 mm bar range from approximately 33 MPa to 45 MPa, depending on the angle, while values between approximately 12 MPa and 18 MPa are measured for 16 mm and between approximately 6 MPa and 15 MPa for 25 mm. At the two early-slip levels, the separation between the two larger diameters closes and partly inverts. For $\tau_{0.01}$, mean values range from approximately 7 MPa to 14 MPa for 8 mm, from approximately 3 MPa to 7 MPa for 16 mm, and from approximately 3 MPa to 9 MPa for 25 mm. The diameter effect is thus most pronounced near the bond capacity.

The magnitude of this effect exceeds the conventional bar-diameter dependence. Bond tests on conventional concrete indicate a reduction of roughly 25 to 40% in bond strength as the bar diameter increases from 10 mm to 50 mm [25], and, for 10 mm and 12 mm bars with identical pull-out failure, the diameter effect was even found to be insignificant, with the mean bond strengths differing by 0.16 MPa, whereas a 16 mm bar in the same programme reached only about 70% of that value because its failure mode changed to splitting [31]. The classical size effect is primarily driven by brittle splitting and diminishes with increasing confinement [32], although a size-dependent bond response has also been reported under confined conditions [33]. Since splitting is suppressed here by the steel confinement rings, only a minor contribution of this mechanism can be expected. The mean $\tau_{\max}$ differs by a factor of about 3.9 between the 8 mm and 25 mm bars and thus exceeds the magnitude of conventional size effects. An additional process-related contribution acting more strongly on larger bars must therefore be present. This contribution is identified with the layer-wise contact-zone manifestation of shadowing below. A comparison with extrusion-based 3D concrete printing shows that the direction of the diameter effect is process-specific: there, the bond strength of bars placed between layers was higher for 10 mm than for 6 mm bars, attributed to the larger absolute rib height of the tested bars, while the porosity at the steel–concrete interface correlated linearly and negatively with the achievable bond [34]. The direction of the diameter effect thus reflects the respective interface formation and rib geometry rather than a universal trend.

By contrast, the inclination angle does not show a consistent monotonic trend over the range from 0° to 90°. Local extrema occur for individual angles, but no systematic ordering can be identified by visual inspection. Hypothesis H2 is therefore not supported by the data, which argues against a governing role of the projected shadowed area.

**Figure 10:**
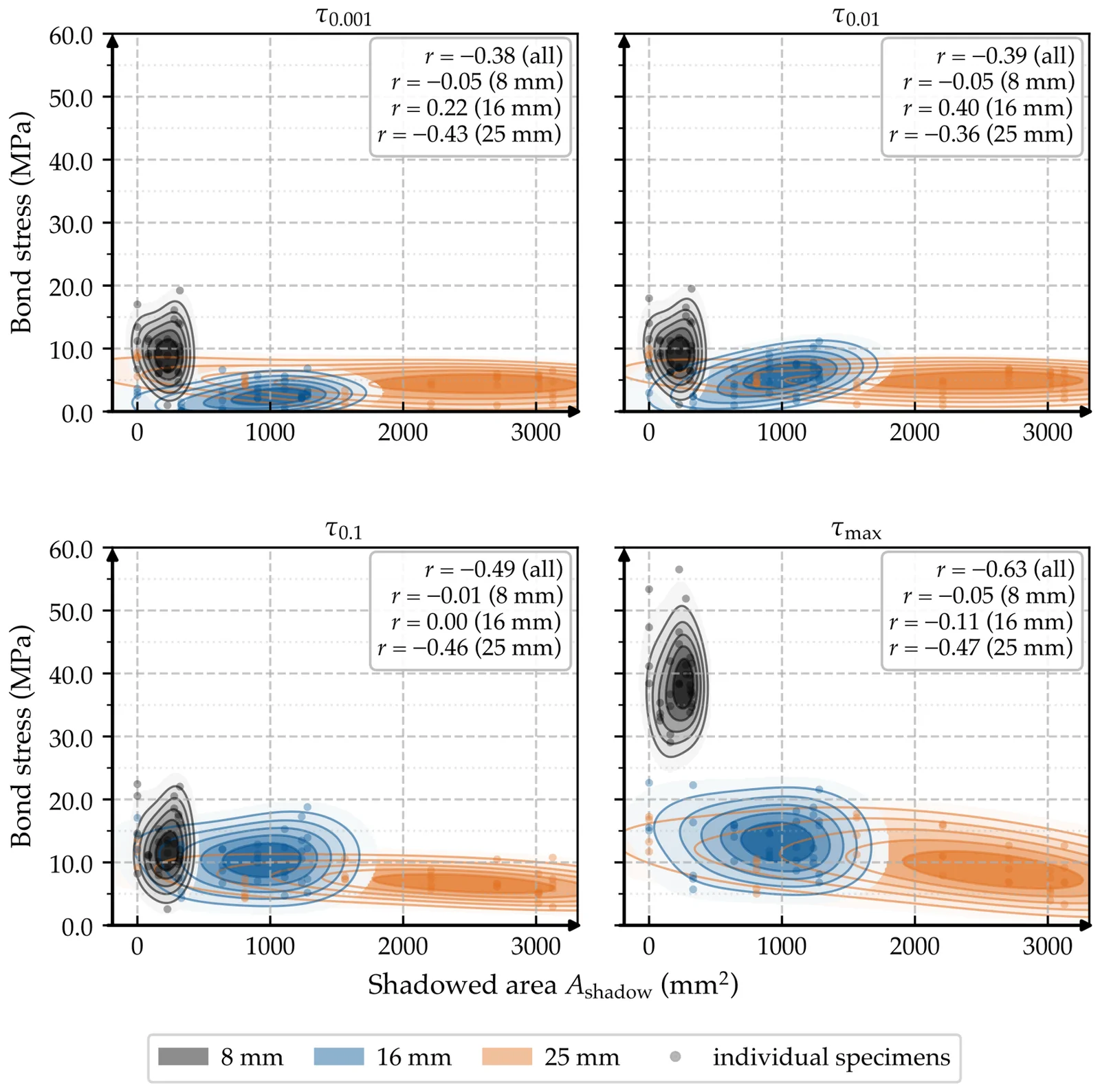
Bond parameters $\tau_{0.001}$, $\tau_{0.01}$, $\tau_{0.1}$, and $\tau_{\max}$ as a function of the projected shadowed area $A_{\mathrm{shadow}}$. Points show individual specimens, and the shaded contours are kernel-density estimates of the per-diameter clusters, evaluated within the respective data range. The correlation coefficients *r* between $A_{\mathrm{shadow}}$ and the bond parameter are given per panel for the pooled data and within each diameter.

The projected-shadow component of the working hypothesis formulated in the introduction predicts a decrease in bond stress with increasing $A_{\mathrm{shadow}}$. Figure 10 shows $\tau_{0.001}$, $\tau_{0.01}$, $\tau_{0.1}$, and $\tau_{\max}$ as a function of $A_{\mathrm{shadow}}$.

The data points are scattered over the entire range of the shadowed area $A_{\mathrm{shadow}}$. Rather than a uniform τ–$A_{\mathrm{shadow}}$ relationship, distinct clusters emerge according to bar diameter. Within each cluster, no clear monotonic structure with $A_{\mathrm{shadow}}$ can be identified. The correlation coefficient between $A_{\mathrm{shadow}}$ and $\tau_{\max}$ is r=−0.63 for the pooled data (n=97), but this value reflects the diameter clustering, since $A_{\mathrm{shadow}}$ scales with $d^{2}$: within the 8 mm and 16 mm series, the correlation vanishes (r=−0.05 and −0.11), and within the 25 mm series a moderate negative correlation remains (r=−0.47, n=33). The same pattern holds at $\tau_{0.1}$ (pooled r=−0.49, within-diameter −0.01, 0.00, and −0.46). The coefficients for all four bond parameters are given in Figure 10. In regions of overlapping shadowed areas, where combinations of different diameters and angles lead to similar values of the shadowed area $A_{\mathrm{shadow}}$, the corresponding bond stresses remain separated by diameter, at $\tau_{\max}$ by up to a factor of about three, and no convergence of bond parameters occurs. A small secondary contribution of $A_{\mathrm{shadow}}$ cannot be ruled out by the present data: a region in the particle bed shielded against deposition can be expected to produce a locally weakened concrete matrix [17,18]. The projected shadowed area, although demonstrably present, is therefore at most a secondary factor, and the dominant mechanism must lie elsewhere.

**Figure 11:**
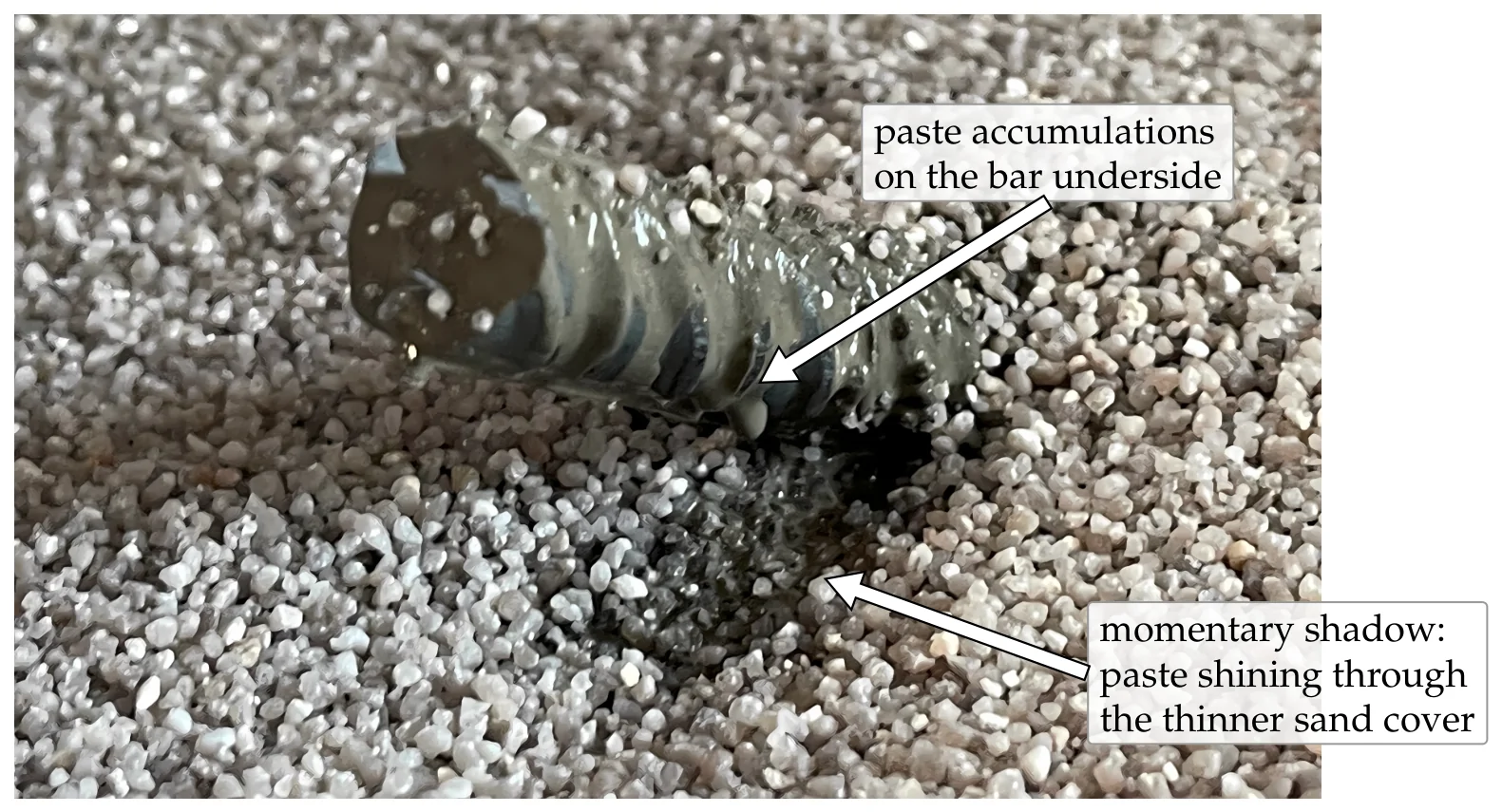
Printed specimen with the momentary shadow directly visible. The protruding bar locally blocks aggregate accumulation from above, so that the sand layer beneath the bar remains thinner than in the surrounding bed. The cement paste of the underlying layer shines through this thin layer and appears as a dark patch beneath the bar. Paste accumulations on the bar underside show that the paste, unlike the aggregate, partially flows around the bar circumference.

The phenomenon is directly visible in the printed specimens. Figure 11 shows a specimen with an aggregate-depleted zone in the particle bed beneath the protruding bar, exactly in the geometric position predicted by Equation (2). During each spreading step, aggregate trickles from above and partially re-fills the region beneath the bar, but the deposited sand layer remains locally thinner there. The dark patch in Figure 11 is the cement paste of the underlying, already intruded layer, which shines through this locally thinner sand cover. Cement paste, unlike the aggregate, is not restricted to deposition from above: it wets the bar, flows around its circumference, and can drip from the underside, as the paste accumulations visible on the bar underside in Figure 11 confirm. This asymmetry between blocked granular transport and liquid paste transport renders the zone beneath the bar paste-rich but aggregate-poor. The limited weight of $A_{\mathrm{shadow}}$ in the bond data is therefore not a question of whether the phenomenon exists, but of how much it contributes relative to other mechanisms. Inserting Equation (3) and the bar surface area within the bond length, $\pi d\;l_{b}$, the shadowed fraction of the bond surface becomes $A_{\mathrm{shadow}}/\left(\pi d\;l_{b}\right)=\left(d\;l_{b}\cos\theta \right)/\left(\pi d\;l_{b}\right)=\cos\theta /\pi$. This ratio is independent of bar diameter and bond length and reaches its maximum of 1/π≈ 32% at θ=0°. The shadowed volume affects only the matrix in a thin lateral strip beneath the bar, while the load-bearing concrete around the rest of the bar circumference remains unaffected. The concrete compression cones that form at the ribs during mechanical interlock bear partly on this weakened strip, but the affected fraction of each cone is small relative to the full bearing area around the bar circumference. The independence of $A_{\mathrm{shadow}}/\left(\pi d\;l_{b}\right)$ from diameter is consistent with the observation that $A_{\mathrm{shadow}}$ does not account for the diameter effect in the data.

The complementary descriptor $A_{\mathrm{contact}}$ predicts a disturbed contact zone along the lower half of the bar surface within the bond length, formed by the sequential overprinting of protruding bar segments. Several process-related effects act simultaneously within this zone. Aggregate particles can trickle and rearrange beneath the bar during layer deposition, and cement paste can flow into the locally less accessible regions. Additional rearrangement processes within the particle bed modify the local packing state. These mechanisms partially counteract a purely geometric shadowing. The affected region should therefore not be interpreted as a material-free void, but as a structurally modified contact zone. A comparable partial filling of the disturbed zone has been reported for paste-coated bar penetrations in 3D concrete printing, where the deliberately applied paste filled the induced voids over the upper 47 to 56% of the penetration depth and increased the flexural capacity of the reinforced sections by about 50% [35]. At the lower part of such penetrations, the surrounding printed material was observed to compact against the bar even without coating, producing high bond [35,36]. How much paste actually flows into the region beneath the bar cannot be quantified from the present data. A geometric bound nonetheless exists. The redistribution length required to reach the region beneath a bar is on the order of the bar radius: about 4 mm, or two to four grain diameters, for the 8 mm bars, but more than 12 mm for the 25 mm bars. Aggregate trickling and paste flow over a few grain diameters can therefore compensate a substantial part of the disturbance beneath the small bars, while the same short-range redistribution leaves most of the zone beneath the large bars unfilled. Because the required transport lengths remain in the range of a few millimetres, this compensation is also consistent with the shape fidelity of the printed specimens, for which paste spreading beyond the intrusion front is limited to a comparable scale [17,18]. Its direct measurement by sectioning and computed tomography is addressed in Section 5. The observation that the diameter effect dominates the response across all slip levels is consistent with a disturbance whose severity increases with *d*, and the absence of a monotonic angle trend matches the constancy of $A_{\mathrm{contact}}$ in θ.

The push-through evaluation normalises the recorded force over the full surface area of the embedded bar (Equation (1)). The geometric size of the bar is thus explicitly accounted for in the evaluation, and a straightforward expectation would be that differences in diameter cancel out and produce comparable bond stresses. The data show a diameter effect nonetheless. This indicates that the geometric bar surface does not contribute uniformly to load transfer: a fraction of the contact area is structurally impaired by the layer-wise manufacturing process, and the impaired fraction, or the disturbance intensity within it, increases with bar diameter. The layer-wise contact-zone disturbance, the second manifestation of shadowing, is therefore the most probable explanation of the observed ordering τ(8 mm)>τ(16 mm)>τ(25 mm) at the developed-interlock and capacity levels ($\tau_{0.1}$ and $\tau_{\max}$). This attribution rests on the mechanical response alone. Direct evidence of the contact zone, from sections or computed tomography, is not available in this study, so the contact-zone mechanism remains a hypothesis (Section 4). An analogous obstacle-size scaling is observed for dense granular flow past a fixed cylinder: in vertical-chute experiments with glass spheres of 3 mm and 6 mm and cylinder diameters of 12.7 mm, 25.4 mm, and 38.1 mm, the drag force on the cylinder grows nearly linearly with the cylinder diameter, decreases with grain size, and is independent of the mean flow velocity over the range studied [37]. This analogue transfers the direction of the size scaling to the SPI contact zone, not its magnitude, because it describes a flowing monodisperse packing rather than a deposited bed. The closest additive-manufacturing analogue points in the same direction: the air-void field forming beneath and around an integrated reinforcement bar in 3D concrete printing enlarges with increasing bar diameter at fixed paste, shown numerically for bar diameters of 6 to 12 mm [38], and the interfacial transition zone around larger aggregate inclusions is more porous at fixed water-to-cement ratio and identical cement [39], a size scaling that can be expected to transfer to cylindrical bars. Normalisation over the full bar surface removes the geometric scaling but not this process-induced inhomogeneity of the contact zone. The observed diameter effect is thus a structural, not a purely geometric, effect of bond formation. These analogues support the direction of the diameter dependence. Its magnitude here, the factor of about 3.9 between the 8 mm and 25 mm bars (Table 2), exceeds what any of them quantifies.

In addition, smaller bars can be more effectively surrounded by aggregate and paste during the deposition process, which eases material flow into the contact zone and can partially compensate for the disturbance. This grain-scale compensation acts on a different length scale from the obstacle-scale disturbance discussed above and does not contradict it: the near-contact packing perturbation scales with the grain size and is hence relatively large, in proportion to the bar circumference, for the smaller bars, whereas the obstacle-induced disturbance scales with the bar diameter. The two effects act in opposite senses. The grain-size scaling of the near-contact perturbation is consistent with wall-effect packing studies at cylindrical boundaries, which report a disturbed layer extending about two particle diameters from the wall [19], with the wall effect that reinforcement exerts on the packing density of concrete, accounted for explicitly in mix-design models [40], with sphere-packing simulations [21] and X-ray computed tomography measurements [22] showing elevated near-wall porosity, and with paste-penetration models for sand packings, which predict easier intrusion with increasing packing porosity and grain size [17,18].

The special case of 90° provides a qualitative consistency check for the contact-zone interpretation. As established in Section 2.4, the mechanism captured by $A_{\mathrm{contact}}$ does not apply at this angle: material accumulates rotationally symmetrically around the vertical bar, and a higher bond would be expected. The data show this trend. The highest or among the highest mean values of $\tau_{\max}$ within each diameter occur at 90°: 45.1 MPa for 8 mm, 17.7 MPa for 16 mm, and 14.8 MPa for 25 mm. The trend is clearest for the 16 mm bar, for which the 90° mean (17.7 MPa) exceeds the next-highest angle mean (14.3 MPa at 60°) by 3.4 MPa. For the 8 mm bar, the 90° value (45.1 MPa) and the 45° value (44.9 MPa) differ by 0.2 MPa, less than the standard deviation of either configuration (6.7 MPa and 7.5 MPa, Table 2). For the 25 mm bar, the 60° value (15.1 MPa) exceeds the 90° value (14.8 MPa) by 0.35 MPa, again less than the standard deviations of 3.0 MPa and 2.7 MPa. The 90° value exceeds the mean of the other six angles by 6.5 MPa for 8 mm, 4.5 MPa for 16 mm, and 5.2 MPa for 25 mm, differences comparable to the standard deviations of the individual configurations.

The angle dependence of the 25 mm series differs from that of the two smaller bars. From 15° to 60°, its mean $\tau_{\max}$ rises monotonically from 6.2 MPa to 15.1 MPa, and the shape of the bond stress–slip curves changes with it. At 0° to 30°, the maximum is reached at a slip of 0.18 to 0.45mm and exceeds $\tau_{0.1}$ by only 15 to 27%, so the bond is exhausted before the rib interlock develops. At 45° and 60°, the maximum is reached at 0.74 mm and 0.97 mm and exceeds $\tau_{0.1}$ by 71% and 83%, the signature of a developed interlock. For the 8 mm bars, the interlock develops at every angle, with $\tau_{\max}$ exceeding $\tau_{0.1}$ by 150 to 350%. This pattern is consistent with the compensation mechanism described above: beneath the small bar, trickling aggregate and paste flow restore the contact zone at every inclination, whereas beneath the large bar they do so only once the projected shadow becomes short enough, which is also reflected in the within-diameter correlation coefficient of r=−0.47 between $A_{\mathrm{shadow}}$ and $\tau_{\max}$ for 25 mm. The 60° value of the 25 mm series is therefore the end point of this rise rather than an isolated extremum, and the interchange of the 16 mm and 25 mm means at 60° follows from it together with the comparatively low 16 mm value at this angle (14.3 MPa). At $\tau_{0.1}$, the interchange does not occur. The rise ends at 75°, where the mean $\tau_{\max}$ is the lowest of the series for 8 mm and 16 mm and the third-lowest for 25 mm. This common feature of all three diameters is not predicted by either descriptor, and no geometric or fabrication-related peculiarity of the 75° configurations was identified.

The segment joints can be assessed as a source of scatter with the segmentation pattern in Table 1. Across the 21 configurations, the mean coefficient of variation of $\tau_{0.1}$ is 37% for the seven continuous configurations, 28 to 30% for two and three segments, 22% for four segments, and 18% for the two five-segment configurations of the 25 mm series, so the scatter at this slip level does not increase with the number of segments. At $\tau_{\max}$, the mean coefficients of variation lie between 16% and 29% without a trend with the number of segments. For the 8 mm series, the mean $\tau_{\max}$ of the continuous bars (0° to 45°, n=18) is 40.8 MPa with a standard deviation of 6.0 MPa, against 37.5 MPa with 6.9 MPa for the segmented bars (60° to 90°, n=13). This comparison is confounded with the angle, so it bounds rather than isolates the joint effect. Within these bounds, the joints do not increase the scatter systematically, and a joint-related reduction of $\tau_{\max}$, if present, is smaller than the angle-to-angle variation of the series. An influence of the adhesive joints and of the segmentation on the bond cannot be ruled out entirely with the present data.

Three influences of the test configuration require examination as potential confounders of this interpretation: the Poisson effect of the push-through loading, the orientation of the layer planes relative to the loading direction, and the radial confinement configuration.

The push-through configuration subjects the bar to axial compression, which causes a lateral expansion of the bar cross-section due to the Poisson effect and thereby increases the radial contact pressure between bar and concrete. In a pull-out test, the bar contracts and the radial pressure decreases. Push-through tests therefore tend to produce higher absolute bond stresses than pull-out tests on otherwise identical specimens: for bars under externally applied lateral pressure, an increased radial pressure at the interface has been shown to raise the bond resistance in those tests by up to 200% [41].

The size of this contribution can be estimated from the elastic constants of the bar. The axial stress in the bar follows from the recorded force as $\sigma =F/(\pi d^{2}/4)$. Expressing *F* through Equation (1) as $F=\tau \;\cdot \;\pi \;\cdot \;d\;\cdot \;l_{b}$ and inserting $l_{b}=5d$ gives

(5) $$\sigma =\frac{\tau \;\cdot \;\pi \;\cdot \;d\;\cdot \;5d}{\pi d^{2}/4}=20\;\tau .$$

The unconstrained lateral expansion of the bar diameter under this stress is $\Delta d=\nu \;\sigma \;d/E_{s}$, with the Poisson ratio ν=0.3 and the elastic modulus $E_{s}=200\;G\mathrm{Pa}$ of reinforcing steel. Within the elastic range, this expansion amounts to approximately 5 to 11μm across the three diameters, below 1.5% of the respective mean rib heights (Table 3, Table 4 and Table 5). The radial pressure that this expansion generates against the surrounding concrete scales linearly with σ and hence with τ itself. Modelling the specimen as a thick-walled cylinder (inner radius d/2, outer radius 40 mm) with an assumed matrix modulus of 20 to 30 GPa gives a radial pressure of approximately 2 to 3.5% of σ, with a geometry factor that varies by less than 20% between the 8 mm and 25 mm configurations (the outer steel ring stiffens this response similarly for all configurations). For the 16 mm and 25 mm series, whose axial stresses remain elastic throughout (at most 354 MPa and 302 MPa, respectively), the Poisson-induced self-confinement amplifies the response by a comparable relative amount and cannot generate their separation. For the 8 mm series, the elastic estimate is a lower bound, since the implied axial stresses exceed the elastic range at $\tau_{\max}$ (see below). In absolute terms, the elastic radial pressure amounts to about 6 to 12 MPa for the 16 mm and 25 mm series at $\tau_{\max}$ and to 18 to 32 MPa for the 8 mm series at its highest $\tau_{\max}$. The magnitude of the resulting change in bond stress can be bounded empirically. In confined pull-out and push-in tests on the same specimen geometry with steel jackets and bar diameters of 5 mm, 12 mm, 18 mm, and 26 mm, the mean peak bond stress of the push-in tests ranged from 83% to 115% of the pull-out value, and no major difference between the two test modes was reported, at axial bar stresses of up to about 700 MPa [33]. The Poisson-induced self-confinement thus changes the absolute bond stress by a fraction on the order of ±15% in that stress range, which covers the 16 mm and 25 mm series at all slip levels and the 8 mm series at $\tau_{0.1}$ (axial stresses of at most 348 MPa). It cannot produce the factor of about 3.9 between the 8 mm and 25 mm series. For the 8 mm series at $\tau_{\max}$, where the implied axial stresses reach 670 to 900 MPa, the bar enters the plastic range, the lateral expansion is no longer bounded by the elastic estimate, and the absolute $\tau_{\max}$ of this series carries an upward contribution that the present data cannot quantify. Tensile tests on the bars used here were not performed, so the tensile strength of 647 to 658 MPa measured for B500B bars in [7] is used as reference.

One restriction on the absolute values follows from Equation (5). With $\sigma =20\;\tau_{\max}$, the 8 mm bars imply axial compressive stresses of 670 to 900 MPa at $\tau_{\max}$, at or above the nominal yield strength of B500B (500 MPa). Local plastic compression of the bar, together with the associated increased lateral expansion, may thus contribute to the high $\tau_{\max}$ recorded for this diameter and to a part of the factor of about 3.9 between the 8 mm and 25 mm series. The ordering of the three diameters is unaffected, since it is already present at $\tau_{0.1}$, where all series remain elastic, but the absolute $\tau_{\max}$ values of the 8 mm series should be read with this restriction in mind.

The layer-wise fabrication introduces a systematic variation of the angle between the loading direction and the layer planes across the test series. At θ=0°, the push-through force acts parallel to the layers, and at θ=90° perpendicular to them. If the SPI matrix exhibited pronounced mechanical anisotropy, this variation would constitute a confounder correlated with the inclination angle. For an SPI concrete with the same aggregate and spread-flow level (400 mm, cf. Section 2.2), produced with a paste of water-to-cement ratio 0.30, no direction dependence of the compressive strength relative to the layer orientation was observed, which was attributed to the load transfer through the directly contacting grain skeleton [2]. The transfer of this observation to the water-to-cement ratio of 0.40 used here is an assumption, since a higher water-to-cement ratio raises the capillary porosity of the paste and can alter the interlayer interface. Direction-dependent strength is documented for extrusion-based printing, where the interfaces between layers and strands make the compressive strength depend on the loading direction [42]. The bond data themselves bound the size of a possible anisotropy effect. A mechanical anisotropy of the matrix would enter the push-through response as a function of the angle between loading direction and layer planes, which changes monotonically from 0° to 90° across the series. The angle means show no monotonic trend at any slip level (Figure 9), so an anisotropy contribution, if present, is smaller than the scatter between adjacent angles and cannot produce the diameter ordering, which holds at $\tau_{0.1}$ and $\tau_{\max}$ at every angle apart from the two exceptions noted above. Matrix anisotropy is therefore not the primary explanation of the diameter effect, but it has not been ruled out experimentally, since the printed material was not tested for direction-dependent strength at the water-to-cement ratio of 0.40.

The third influence is the radial confinement configuration. The concrete cover between the bar surface and the outer specimen surface follows directly from the specimen geometry as (80−d)/2 in mm, owing to the constant outer specimen diameter of 80 mm. The cover and its ratio to the bar diameter therefore vary across the test series, from 36 mm (ratio ≈4.5) for 8 mm to 27.5 mm (ratio ≈1.1) for 25 mm. The steel confinement rings suppress splitting at all diameters, so that the measured response is governed by bond. If the shift in radial stiffness towards the steel ring at the larger diameters governed the diameter effect, higher bond stresses would be expected at the larger diameters, where the stiff ring lies closest to the bar. The data show the opposite trend. The confinement configuration therefore cannot account for the observed diameter dependence.

The phenomenology described above has a parallel in the top-bar (or top-cast) effect in conventionally cast reinforced concrete. In gravity-cast members, horizontally placed bars near the top of the cast section experience reduced bond owing to the settlement of the fresh concrete and the accumulation of bleed water beneath the bar [43,44,45]. The reduction increases with casting depth and with the water content of the mixture [44], and it has also been reported for self-compacting concretes (SCC), in which the local bond strength of top-cast bars was about 20% lower than in comparable normal concrete [46]. For SCC, the static stability of the mixture has been identified as the governing parameter, with a locally weaker interfacial zone of reduced modulus and micro-strength reported beneath the bar, although this reduction is less pronounced than for vibrated concrete [47]. In the present data, the bar diameter takes over the role that the casting position plays in these studies, since the severity of the underside disturbance grows with the obstacle size. Reported bond reductions range from approximately zero to more than 50% depending on specimen geometry, bond length, and the slip range considered, magnitudes similar to the relative differences observed here between the 8 mm and 25 mm bars [48].

The parallel is phenomenological only: in gravity-cast concrete, the underside disturbance is driven by settlement and bleed water in a fluid concrete, promoted in particular by vibration during compaction. In the SPI process considered here, no fluid concrete bath exists. The matrix is built up layer-wise from a dry particle bed selectively infiltrated by paste, and the bar is encased by repeated deposition events rather than floated through a sedimenting medium. The disturbance described by $A_{\mathrm{contact}}$ arises from this layer-wise build-up combined with the wall-effect-related packing perturbation at the cylindrical inclusion. For this reason, the empirical bond-condition factors that design codes for cast-in-place concrete assign to top-cast bars must not be transferred to SPI members. Any reduction factor for reinforcement embedded during printing has to be derived from tests on printed specimens, and the diameter-dependent reductions reported here refer to the boundary conditions of this programme (Section 4).

In addition to the test configuration, the bars themselves differ in rib geometry, which is a further candidate explanation for the diameter ordering. The relative rib area $f_{R}$ was evaluated for all three diameters from the rib parameters defined in DIN EN ISO 15630-1 [49], measured on two ribs per diameter and evaluated with the Simpson formula of that standard, and amounts to $f_{R}=0.079$, 0.094, and 0.069 for the 8 mm, 16 mm, and 25 mm bars, respectively. All values exceed the characteristic values required by DIN 488-2 [50] (0.045 for 8 mm, 0.056 for 16 mm and 25 mm). The measurements are summarised in Table 3, Table 4 and Table 5. For the 25 mm bar, the rib heights and the flank angle were re-measured by laser line scanning after the rib-measuring device had returned an implausible profile for one rib row. Table 5 reports the laser-scan values for these quantities. In these tables, $h_{m}$ is the transverse-rib height at mid-length and $h_{1/4}$, $h_{3/4}$ the rib heights at the quarter points (denoted $a_{m}$, $a_{1/4}$, and $a_{3/4}$ in the standard), *c* the rib spacing, α and β the rib flank and inclination angles, *e* the rib-row spacing, *b* the rib head width, and *l* the rib length. In the mean row, *e* is reported as the sum over the two rib rows, as this sum enters the $f_{R}$ evaluation, whereas the other mean-row entries are arithmetic means.

The standard prescribes at least three ribs per rib row, whereas two ribs per diameter were assessed here, so these values are indicative rather than a conformity assessment.

A consistent monotonic relationship between $f_{R}$ and the bond parameters is not evident. If the rib geometry governed the response, the 16 mm bars with the highest $f_{R}$ (0.094) would be expected to reach the highest bond stresses. Instead, the 8 mm bars reach the highest bond stresses with an intermediate $f_{R}$ (0.079), the 16 mm bars lie in between, and the 25 mm bars combine the smallest $f_{R}$ (0.069) with the lowest bond stresses. The effect of $f_{R}$ on bond is reported to depend on the confinement and on the considered slip range: without confinement, bond strength is largely independent of the deformation pattern, whereas confined bars show bond strengths increasing with $f_{R}$ [51]. In a parametric study, bond stress showed small sensitivity to $f_{R}$ between 0.10 and 0.15 and a strong increase above 0.16, a range above the values measured here [52], and a combined effect of rib width and clear rib spacing has been reported [53]. The lack of a clear $f_{R}$ correlation despite the confined configuration indicates that local rib geometry is secondary to the process-induced effects along the contact zone in the SPI configuration considered here.

## 4. Limitations

The conclusions are bounded by the conditions of the test programme. All specimens were produced with a single vertical protrusion of h=40 mm. The accumulated shadowed area in Equation (3) is independent of *h*, but the momentary shadow in Equation (2) and the intensity of the contact-zone disturbance can be expected to change with *h*, so the results describe the bond at this one boundary condition, and other protrusions (for example 50 mm) require their own test series. A single paste and aggregate system was used (water-to-cement ratio 0.40, quartz sand 1.0 to 2.2 mm), and the transfer of the direction-independence of the compressive strength from a water-to-cement ratio of 0.30 rests on the argument given in Section 3 rather than on measurements at 0.40, so matrix anisotropy is not the primary explanation of the diameter effect but has not been ruled out experimentally. The bars were segmented for most inclined configurations. The joint effect is bounded by the partial control in Section 3 but not isolated, so an influence of the adhesive joints cannot be ruled out entirely. The contact zone was not characterised directly, so $A_{\mathrm{contact}}$ remains a geometric working assumption whose extent and intensity have not been measured, and the attribution of the diameter effect to it is the most probable interpretation rather than a demonstrated mechanism. It is a hypothesis until sections or computed tomography of the contact zone have been evaluated. The replicate numbers of three to five per configuration limit the resolution of angle contrasts: differences smaller than the standard deviations of the individual configurations (Table 2) cannot be resolved. The push-through loading adds a Poisson-induced radial pressure, which is bounded to about ±15% in the elastic range but not for the 8 mm series at $\tau_{\max}$. The rib geometry was assessed on two ribs per diameter.

## 5. Conclusions and Outlook

This study investigates the bond behaviour at protruding reinforcement bars in the Selective Paste Intrusion (SPI) process for varying bar diameters (8 mm, 16 mm, and 25 mm) and inclination angles (0° to 90° in 15° steps). The bar diameter dominates the bond response, most pronounced at the developed-interlock and capacity levels, and the magnitude of the diameter effect exceeds what a conventional pull-out comparison would predict. The inclination angle produces no monotonic trend, with individual angle contrasts remaining largely within the experimental scatter, and plays at most a minor role compared to the diameter. Of the two hypotheses formulated in the introduction, H1 (diameter) is confirmed and H2 (angle) is rejected.

The projected shadowed area $A_{\mathrm{shadow}}$ is at most a secondary factor. Combinations of diameter and angle that lead to similar shadowed areas produce bond parameters separated by diameter, and a consistent τ–$A_{\mathrm{shadow}}$ structure is not observed. The layer-wise contact-zone descriptor $A_{\mathrm{contact}}$ is the most probable interpretation of the data. It remains a hypothesis until the contact zone has been verified directly. It localises the affected region on the lower half of the bar surface within the bond length, where the disturbance is built up by the sequential overprinting of protruding bar segments. The descriptor is independent of inclination angle for 0°≤θ<90° and ceases to apply at θ=90°. The observed elevation of bond capacity at 90° is qualitatively consistent with this loss of the contact-zone mechanism. The normalisation of bond stress over the full bar surface removes the purely geometric scaling but does not capture the process-induced inhomogeneity of the contact zone, so that the observed diameter effect is best read as a structural rather than as a purely geometric effect of bond formation. The required increase of the contact-zone disturbance with bar diameter is consistent with the diameter scaling of granular-obstacle disturbances [37], of air-void formation beneath and around integrated bars [38], and of interfacial porosity around embedded inclusions [39], while its magnitude in the present data remains to be verified directly. The phenomenology has a parallel in the conventional top-bar effect, but the causal driver is different. The disturbance in the SPI case arises from layer-wise deposition combined with obstacle-scale and packing-related disturbances, not from sedimentation in a fluid concrete bath.

Future work should focus on a more direct characterisation of the contact zone. Sectioning of specimens along the bar axis and X-ray computed tomography can visualise the underside contact zone directly and quantify whether the process-induced void volume remains partially unfilled or is compensated by paste intrusion. The comparison of the upper and lower contact zone at 0° and 15°, where the projected shadow is largest, is the first step. Process parameters that govern deposition geometry and intrusion behaviour, such as layer thickness and the aggregate-to-bar diameter ratio, are candidates for systematic variation, since the contact-zone interpretation suggests that they directly modulate the affected region. Coupled experimental and numerical modelling of paste intrusion in disturbed packings beneath cylindrical inclusions would close the remaining gap between the layer-wise contact-zone interpretation and a fully mechanistically validated bond model for reinforced SPI components. A variation of the protrusion height (for example 20 mm, 40 mm, and 50 mm at one diameter and two angles) would show whether the contact-zone disturbance scales with *h* and would place the present results within the process window of [3], and a separate test series with continuous and segmented bars of identical geometry is required to isolate the joint effect.

## Figures and Tables

**Table 1:** Maximum effective bar length $L_{\mathrm{eff}}=\left(h-d\cos\theta \right)/\sin\left(\theta \right)$ above the particle bed at h=40 mm vertical protrusion, number of segments $n_{s}$, and as-built segment lengths for all diameter–angle combinations. The required total length is the bond length 5d plus 10 mm of free bar end for the displacement sensor (±2 mm). Segments were cut to millimetre precision within a tolerance of ±2 mm. All values refer to the nominal bar diameter. The effect of the rib height is quantified in the text. Rounding to full millimetres and the continuous 45° configuration of the 8 mm series can marginally exceed the nominal $L_{\mathrm{eff}}$ (by up to 1.4 mm), and hence the momentary protrusion, within this tolerance. The last segment includes the free bar end.

*d* (mm)	Required Length (mm)	θ (°)	$L_{\mathrm{eff}}$ (mm)	$n_{s}$	Segment Lengths (mm)
8	50	0°	— ^a^	1	continuous
		15°	124.7	1	continuous
		30°	66.1	1	continuous
		45°	48.6	1	continuous
		60°	41.6	2	40+10
		75°	39.3	2	40+10
		90°	40.0	2	40+10
16	90	0°	— ^a^	1	continuous
		15°	94.8	1	continuous
		30°	52.3	2	50+40
		45°	40.6	3	40+40+10
		60°	37.0	3	37+37+16
		75°	37.1	3	37+37+16
		90°	40.0	3	40+40+10
25	135	0°	— ^a^	1	continuous
		15°	61.2	3	60+60+15
		30°	36.7	4	35+35+35+30
		45°	31.6	5	30+30+30+30+15
		60°	31.8	5	30+30+30+30+15
		75°	34.7	4	35+35+35+30
		90°	40.0	4	40+40+40+15

^a^ At 0°, the bar lies parallel to the particle bed at a constant offset of 40 mm. Length is then governed by the bond length plus the bar ends for load application and displacement measurement.

**Table 2:** Mean bond stresses ± standard deviations in MPa at the four characteristic slip levels for all diameter–angle combinations (*n*: number of evaluated specimens per configuration).

*d* (mm)	θ	*n*	$\tau_{0.001}$	$\tau_{0.01}$	$\tau_{0.1}$	$\tau_{\max}$
8	0°	5	12.2±4.5	12.4±4.5	14.3±4.8	37.0±2.7
	15°	5	8.1±3.7	8.4±3.8	12.4±4.7	38.3±3.2
	30°	3	13.8±2.8	14.2±3.0	17.4±3.9	44.4±6.6
	45°	5	7.0±3.9	7.3±4.0	9.9±4.5	44.9±7.5
	60°	5	8.0±2.2	8.2±2.2	11.1±3.1	34.6±5.2
	75°	4	10.2±1.4	10.2±1.3	10.7±0.9	33.6±1.2
	90°	4	12.3±4.6	12.6±4.7	16.5±6.5	45.1±6.7
16	0°	5	3.1±2.4	6.8±2.5	11.5±5.4	13.8±6.2
	15°	5	2.3±0.3	6.8±2.4	12.0±3.8	13.8±4.0
	30°	5	2.9±2.5	5.3±3.0	9.7±4.2	13.2±4.0
	45°	5	3.4±1.4	6.2±2.4	10.4±2.1	12.6±1.9
	60°	5	2.4±2.7	3.7±2.2	9.8±2.3	14.3±2.4
	75°	5	0.4±0.6	2.6±2.4	8.4±3.9	11.9±7.1
	90°	3	3.1±0.5	5.5±2.4	14.5±2.5	17.7±4.2
25	0°	5	3.5±2.1	4.4±1.9	6.7±2.8	7.7±3.4
	15°	5	5.0±0.9	5.3±1.0	5.4±1.7	6.2±1.9
	30°	5	4.4±2.0	5.9±1.0	7.2±1.8	9.2±2.5
	45°	5	2.6±2.2	3.1±1.7	7.2±1.2	12.3±3.5
	60°	4	2.3±1.1	3.5±1.6	8.3±1.6	15.1±3.0
	75°	5	4.8±0.6	4.9±0.6	5.6±1.4	8.1±2.5
	90°	4	8.0±1.7	8.6±1.4	11.7±1.9	14.8±2.7

**Table 3:** Geometric rib parameters for d=8mm.

	*d* (mm)	$h_{m}$ (mm)	$h_{1/4}$ (mm)	$h_{3/4}$ (mm)	*c* (mm)	α (°)	β (°)	*e* (mm)	*b* (mm)	*l* (mm)	$f_{R}$ (–)
1	8.0	0.87	0.55	0.61	5.7	47	58	1.38	1.2	13.2	0.079
2		0.78	0.50	0.51	5.6	47	59	1.38	1.0	13.1	
Mean		0.83	0.53	0.56	5.7	47	59	2.76 ^a^	1.10	13.1	

^a^ Sum over the two rib rows. This sum enters the $f_{R}$ evaluation.

**Table 4:** Geometric rib parameters for d=16mm.

	*d* (mm)	$h_{m}$ (mm)	$h_{1/4}$ (mm)	$h_{3/4}$ (mm)	*c* (mm)	α (°)	β (°)	*e* (mm)	*b* (mm)	*l* (mm)	$f_{R}$ (–)
1	16.0	1.42	1.33	1.11	9.6	46	55	2.36	1.9	27.8	0.094
2		1.26	1.12	1.08	9.7	45	57	2.39	1.8	27.1	
Mean		1.34	1.23	1.10	9.7	46	56	4.75 ^a^	1.85	27.5	

^a^ Sum over the two rib rows. This sum enters the $f_{R}$ evaluation.

**Table 5:** Geometric rib parameters for d=25mm.

	*d* (mm)	$h_{m}$ (mm)	$h_{1/4}$ (mm)	$h_{3/4}$ (mm)	*c* (mm)	α (°)	β (°)	*e* (mm)	*b* (mm)	*l* (mm)	$f_{R}$ (–)
1	25.0	1.94	1.21	1.29	15.0	36	60	3.43	2.6	41.5	0.069
2		1.88	1.24	1.17	14.9	36	58	3.26	2.9	42.4	
Mean		1.91	1.22	1.23	15.0	36	59	6.69 ^a^	2.75	41.9	

^a^ Sum over the two rib rows. This sum enters the $f_{R}$ evaluation.

## Data Availability

The data supporting the results of this study are available on Zenodo at https://doi.org/10.5281/zenodo.22019164. The data are currently under embargo until the publication of the associated article. Upon publication, the data will be publicly accessible under the Creative Commons Attribution 4.0 International (CC-BY 4.0) license.

## Appendix A. Excluded Tests

**Table A1:** Tests excluded from the evaluation with the exclusion criterion in Section 2.8, the recorded force *F*, the bond stress τ calculated from it with Equation (1), the mean ± standard deviation of the retained replicates at $\tau_{\max}$ (group mean), and the deviation of the excluded value from that mean. Criteria: (i) no usable slip signal during the load rise (displacement transducer failed, test stopped), so *F* is the highest force of the record, reached at the stop, and not a maximum bond force. (ii) Displacement offset at the start of loading, the force record is complete and a maximum was reached, $F=F_{\max}$.

Test	Configuration	Criterion	*F* (kN)	τ (MPa)	Group Mean (MPa)	Deviation (%)
8-30-4	8 mm, 30°	(i)	70.3	69.9	44.4±6.6	+58
8-30-5	8 mm, 30°	(i)	50.1	49.8	44.4±6.6	+12
8-90-1	8 mm, 90°	(i)	40.6	40.4	45.1±6.7	−10
16-90-4	16 mm, 90°	(i)	64.7	16.1	17.7±4.2	−9
16-90-5	16 mm, 90°	(ii)	86.0	21.4	17.7±4.2	+21
25-60-2	25 mm, 60°	(ii)	233.5	23.8	15.1±3.0	+58
25-90-3	25 mm, 90°	(i)	102.2	10.4	14.8±2.7	−29
